# Supplementary material for: Research on multi-objective hierarchical site selection coverage of fire station
Source: PLoS One. 2024 Dec 23;19(12):e0309731. doi: 10.1371/journal.pone.0309731 (PMC11666039; doi:10.1371/journal.pone.0309731)
Supplement: Attachment — Submitted filename: PONE-D-24-13730_Suggestions and comments.pdf [file pone.0309731.s002.pdf]

Click here to access/download;Manuscript;Manuscript.docx

Research on multi-objective hierarchical site selection coverage of fire station

HE Junjie[1¶], GUAN Xin[2¶*], LU Houjun[2¶], YANG Juntao[1¶]

1 Shanghai Fire Research Institute Of MEM, Shanghai, China;

2 Logistics Engineering College,Shanghai Maritime University, Shanghai, China

* Corresponding author

E-mail: 202230210302@stu.shmtu.edu.cn

Abstract

The fire station location has important theoretical and practical value. In order to study the multi-objective siting problem of fire stations, we divided demand areas and fire stations into three levels to form a hierarchical emergency coverage network covering fire risk areas in a comprehensive way. Secondly, the nodes of the original location of the fire station were added to the set of nodes of the planned construction of the fire station. By introducing the coverage attenuation function, the multi-objective level fire station location model covering the maximum fire risk value and the minimum construction cost of the fire station was established. The epsilon constraint method was used to process the multi-objective model, and the genetic algorithm was designed to solve the model. Finally, taking an urban area as a specific example, the sensitivity analysis was used to discuss the influence of different parameters on the maximum fire risk value covered by fire stations, and finally, the compromise coordination site selection scheme of fire stations was given, which proved the feasibility of the proposed model.

Keywords: public safety; fire station level site selection; genetic algorithm; epsilon constraint method

Introduction

With the rapid increase of high-risk places such as high-rise buildings, densely populated areas, and flammable constructions, the fire safety and stability of urban environments are facing great challenges. At present, most of the domestic planning for the location of fire stations is only divided into jurisdictions by administrative areas, major roads, etc., the boundaries of the jurisdictions are not obvious, and there is a lack of overall systematic consideration of the distribution of rescue forces, which leads to an imbalance of workload in the face of the existing fire stations in the case of fire, and the scope of some firefighting areas of responsibility is too large, so that the firefighting forces can not reach the scene promptly, and this has brought great difficulties to firefighting and rescue efforts. Therefore, it is necessary to stratify the fire stations, re-plan the coverage area of fire stations according to different levels, and build new fire stations to meet the needs of urban emergency rescue and improve the efficiency of the urban emergency rescue system. However, the construction of

new fire stations undoubtedly requires a huge amount of financial support, but if we can consider retaining some of the original fire stations, and setting up fire stations in the peripheral areas of the city where the firefighting force is insufficient, so that the siting plan can cover all the fire risk zones, and coordinate the functions and rescue forces of the responsibility areas of each fire station. Therefore, how to scientifically plan the location of fire stations on the basis of considering the layering of fire stations is an important issue currently faced.

Currently, site optimization of fire stations has become a popular research topic among scholars. Studies on fire station siting and layout mainly cover the areas of cities [1-5], chemical industry parks [6-8], airports [9-10], etc. For urban fire station siting, Deng et al. [11] proposed a fire station service zoning model by using urban road network. Hu et al.[12] used minimizing the average response time as the optimization objective to calculate the desired response time and the desired service time of each district, and based on this, they studied the fire station responsibility area division model. However, there are some limitations in these above studies that utilize a single objective for site selection because the site selection of the fire station involves basic influencing factors such as economy, time, distance, coverage, etc., and there is uncertainty among these influencing factors, and even mutual constraints among the factors. So the multi-objective planning theory has good simple adaptability to solve the problem of site selection, which can better solve the problem, and the method is currently more widely used in emergency facilities siting[13], the scheduling of the vehicle[14], and so on. In 1998, MASOOD et al.[15] first used multi-objective planning theory to analyze the siting problem of fire stations, laying the foundation for future research on choosing multi-objective models to solve the siting problem. In 2007, Zhang et al.[16] introduced the theoretical idea of fuzzy mathematics to develop a multi-objective site selection model based on fuzzy mean, which takes into account factors such as economy, time and distance. In 2021, Shang[17] proposed to establish an urban fire risk assessment index system from four aspects: urban area information, urban fire risk sources, historical fire disaster, and fire rescue strength, and set different fire station response times based on the assessment results, and optimized the layout of urban fire stations with the help of GIS software. In 2022, Yu et al.[18] proposed the model intersection method based on maximizing the coverage model and minimizing the number of facility points model to calculate the coverage of fire risk points of interest (POI) and the area coverage of the newly planned sites. Huo et al.[19] used ArcGIS to simulate the distribution of fire potential and graded to establish a linkage fire station location optimization model based on demand level and distance loss.

Among the related research on the hierarchical siting model, Moore et al.[20] firstly studied the problem of service facility tiering and constructed the multi-objective siting model with minimum service cost and maximum service efficiency, and solved the solution to verify that the final siting plan could meet the service requirements of customers of all tiers. Serra et al.[21] maximized the number of demand points served by each level of facility in the objective function while using the weighted summation method for the solution. Wang et al.[22] proposed the concept

of hierarchical service facilities for managing services with multilevel service scopes, and developed a multilevel greedy heuristic algorithm to demonstrate that a hierarchical service facility is an effective tool for optimizing the allocation of service resources and balancing the public services in different sub-districts of a residential area. Xiao et al.[23] constructed a maximum coverage site selection model by considering layer characteristics and coverage decay function.

In summary, it can be seen that there are fewer empirical studies on the siting problem of emergency facilities by scholars. Furthermore, in the study of fire station siting planning, less literature considers the characteristics of hierarchical emergency fire stations and hierarchical fire risks in the demand area. Therefore, the author intends to spatially analyze the fire station rescue area based on the fire station planning research proposed by various scholars. The emergency fire station is divided into the primary fire station, the secondary fire station and the tertiary fire station to construct the fire station siting model of the three levels, so as to realize the scientific and reasonable siting of the urban fire station. This can provide some academic help to the current theoretical research and solutions for the siting of fire stations.

Multi-objective Hierarchical Coverage Site Selection Model for Fire Stations

Description of the Problem

From the current situation of urban emergency management today, it can be seen that the handling of fire emergencies and sudden accidents in cities is characterized by a certain degree of hierarchy. Based on the establishment of fire stations in the current literature at home and abroad, a hierarchical service network of fire stations is proposed. The hierarchy of fire stations can be divided into three types of fire stations: grade I , grade II and grade III based on the area, staffing, rescue equipment in the construction standard of fire stations.

The nested relationship between the three types of fire stations is set as follows: the highest level of the fire station has the capacity and coverage to cover all three levels of demand; the secondary fire station also covers medium and low risk demand; and the primary fire station covers the area with the lowest level of risk.

Different fire risk areas have different needs for different levels of fire stations, and this paper categorizes fire risk areas into low-risk areas, medium-risk areas and high-risk areas. High-risk areas, because of their high-risk level, can only be served by tertiary fire stations with strong rescue capabilities and specialized rescue personnel and rescue equipment in the event of a fire, while low-risk areas, because of their own relatively low risk in the area, are provided with rescue services by primary fire stations. The emergency rescue service network is shown in Figure 1 below.

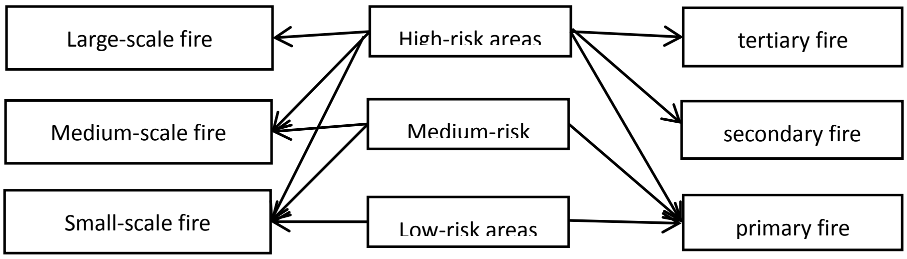

Fig 1. Schematic diagram of the structure of the emergency rescue service network

The emergency services network in the figure above demonstrates how the primary, secondary and tertiary fire stations present a complementary effect in rescue services. In the process of site selection, for the three levels of fire stations to provide accurate and comprehensive coverage within the region, it is necessary to analyze the level capacity of the existing fire stations and planned fire stations, as well as the degree of risk level at the point of demand, in order to scientifically and efficiently optimize the layout of the existing fire station coverage and to cover more fire risk levels.

Based on the fire station rescue service network, the idea of fire station hierarchy is proposed, schematically shown in Figure 2. When a fire occurs at a demand point in a selected area within a city, the nearest fire station is required to provide fire suppression services to reduce the damage and impact of the fire. Two problems need to be solved: first, how to arrange for the closest fire station that is within the capability coverage to provide fire fighting services for the demand point when the risk level of the demand point is determined; and second, after determining the minimum number of fire stations covering the scope of the selected area, how to solve for the siting configuration of the minimum number of new fire stations required on the basis of retaining some or all of the existing fire stations.

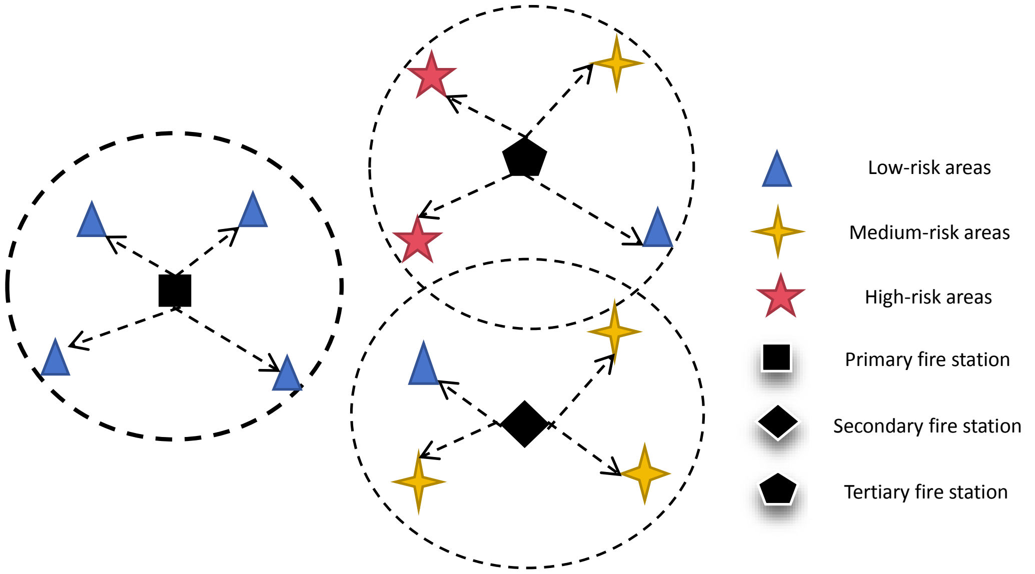

Fig 2. Schematic diagram of the siting of a tiered fire station

Based on the above considerations, the following hypotheses are proposed for constructing a multi-objective hierarchical siting model for fire stations that considers fire risk and capacity constraints:

(1) The distances between demand points and fire station candidate points in the model are Euclidean distances.

(2) Demand points are known at each level and both facility and demand points are discrete.

(3) Fire station facility points can provide emergency response services to multiple demand points.

(4) Different levels of service can be provided to multiple demand points within the capacity of the facility point.

(5) Service satisfaction decreases as the radius of coverage of the fire station to the point of demand increases.

Definition of variables and parameters

I represents the set of demand points, $\forall i \in I$. J represents the set of fire station planning candidates, $\forall j \in J$. K denotes the level set of demand points, $K = \{1,2,3\}$. H denotes the set of fire station levels, $H = \{1,2,3\}$. k_i indicates the level corresponding to demand point i, $k_i \in K$. h_j indicates the level corresponding to facility point j, $h_j \in H$. a_i denotes the value of fire risk at demand point i. d_{ij} means the distance from i to j. S_j is the maximum distance that the demand point is covered by the fire station j, $d_{ij} \leq S_j$. $f_{ij}(d_{ij})$ is denoted as the coverage provided by facility point j for demand point i. q indicates the number of existing fire stations. p indicates the number of fire stations that need to be built. c_j denotes the capacity of the facility point j. Ω indicates the collection of existing fire stations.

Coverage attenuation function

The classic siting model has the parameter of the radius of coverage of a facility point. Any demand point within the coverage radius can be served by a fire station facility point in the event of a fire, but when a demand point is outside the coverage radius it is considered to be unable to cover that demand point. It is assumed that the coverage area of a city fire station is a circle with a radius of 4 kilometers. If the location of a risk area is at a distance of 4.01 kilometers from the center of the circle, it is also treated in the siting model as if it cannot be covered. However, in real life, even if some demand points are not within the radius of the coverage area of the fire station, they can still be covered by the fire station. The traditional site selection coverage model is too strict in determining coverage or non-coverage. Therefore, in this paper, the coverage decay function is introduced. Equation (1) relaxes this concept to make the fire station coverage model more realistic.

$$f_{ij}(d_{ij}) = \begin{cases} 1 & , d_{ij} \leq s_j \\ \left[1 - \dfrac{d_{ij} - s_j}{\max d_{ij} - s_j}\right]^2 & , d_{ij} > s_j \end{cases} \tag{1}$$

Different levels of fire risk areas in urban areas have different quality requirements for fire emergency response services, and the ability of fire stations to meet a wider range of emergency response services is also a key issue in the site selection model. The idea of the coverage decay function is expressed as follows: demand points beyond the coverage of the facility point are still covered, but the coverage service capability of the emergency facility point for the fire risk area is slowly decreasing as the distance from the facility point to the fire risk area increases. When the emergency facility point to the fire risk level area is within the coverage

capacity of the facility point, the coverage of the fire station to the demand point is 1. When the distance from the fire risk area to the facility point is far beyond the coverage of the facility point, the rescue service capacity of the facility point tends to 0, as shown in Figure 3 below.

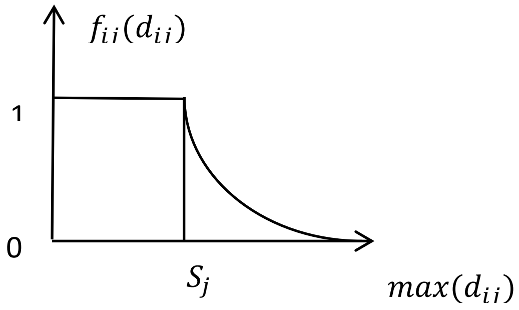

Fig 3. Plot of coverage attenuation function

Construction of the Mathematical Model

In response to the problem posed by the problem description, the research object is networked and the model is built as shown below, taking into account constraints such as the hierarchy of fire stations:

$$Z = \max \sum_{i \in I} \sum_{j \in J \cup \Omega} a_i f_{ij}\left(d_{ij}\right) x_{ij} \quad (2)$$

$$\min \sum_{j \in J} y_j \quad (3)$$

$$\min \sum_{j \in \Omega} y_j \quad (4)$$

S.t.

$$\sum_{j \in J \cup \Omega} x_{ij} = 1, \forall i \in I \quad (5)$$

$$x_{ij} \leq y_j, \forall i \in I, \forall j \in J \cup \Omega \quad (6)$$

$$\sum_{j \in J \cap \Omega} y_j = p \quad (7)$$

$$\sum_{i \in I} x_{ij} a_i \leq c_j, \forall j \in J \cup \Omega \quad (8)$$

$$x_{ij} k_i \leq h_j y_j, \forall i \in I, \forall j \in J \cup \Omega \quad (9)$$

$$x_{ij} = \begin{cases} 1, \text{Facility point } j \text{ provides coverage for demand point } i \\ 0, \text{Facility point } j \text{ does not provide coverage for demand point } i \end{cases} \quad (10)$$

$$y_j = \begin{cases} 1, \text{Facility point } j \text{ is selected} \\ 0, \text{Facility point } j \text{ is not selected} \end{cases} \quad (11)$$

Equation (1) represents the coverage decay function for facility point j providing service to demand point i. Equation (2) represents the fire risk weighted values for maximizing coverage. Equation (3) indicates the minimum number of new facility

sites. Equation (4) indicates that the largest number of existing fire stations will be retained. Equation (5) indicates that each demand point is provided coverage by a fire station. Constraint (6) indicates that service is provided only when fire station j is selected. Equation (7) indicates the number of local fire stations planned to be built. Constraint (8) indicates that the fire risk covered by the fire station does not exceed the maximum capacity. Constraint (9) indicates that demand point i can be served by fire stations of the appropriate level or higher level. Equation (10) and (11) defines two decision variables.

In this paper, the constraint method is chosen to process the multi-objectives, and the objective function (2) in the model is retained as the decision objective of the final model. The objective functions (3) and (4) are treated as constraints of equations (12) and (13) so that they are constrained to two acceptable values A and B, respectively, and then they are substituted into the model to solve the problem, and a set of trade-off solutions are obtained by continuously adjusting the values of A and B.

$$\sum_{j\in J} y_j \leq A \tag{12}$$

$$\sum_{j\in \Omega} y_j \geq B \tag{13}$$

Methods and Steps for Solving the Mathematical Model

The site selection problem is one of the NP-hard problems, with the increasing size of the demand points and candidate points divided in the selection area, it will take a lot of time to solve the model, and the heuristic algorithm can be more flexible, efficient, and fast to find the optimal solution of the model. Therefore, this paper chooses a genetic algorithm to solve the multi-objective hierarchical fire station siting model on the basis of the constraint method to deal with the target, and the algorithm is designed as follows:

(1)Coding: For the optimization model above, the constraints of the hierarchical fire station siting model are handled by applying a dual structure coding, which avoids the generation of invalid individuals. The chromosome is composed of two parts, the variable code and the additional code, as shown in Figure 4 below (with 6 candidate points). The upper row indicates a candidate point for a fire station and the lower row indicates whether the fire station is selected or not and the assignment of demand points must satisfy the constraints of the model.

The additional code	5	2	4	6	1	3
The variable code	1	0	0	1	1	0

Fig 4. Chromosome coding

(2)Population initialization: the initial population is randomly generated and each fire station candidate has a probability of being selected as a fire station. The population size N is set to 200.

(3)Adaptation value function: observing the model gives that for each chromosome, a_i is known and the value of $f_{ij}(d_{ij})$ can be derived from d_{ij}. In addition, equation (2) is for the maximum value, so the fitness of the chromosome is

defined as the inverse of the objective function:

$$f(x_i) = \frac{1}{Z} \tag{14}$$

(4)Selection: based on the previous step, individuals with large values of the fitness function are calculated and these superior chromosomes are selected to produce the next generation of offspring, while the remaining individuals are operated according to the following roulette mechanism. Separately, the fitness value of each individual is calculated as f. Where I is the population size, the probability of inheriting to the next individual is $P_{(x_i)}$, and the cumulative probability of each individual is $Q_{(i)}$. The two probability formulas are shown in equations (15) and (16). Generate a uniformly distributed random number r in the interval (0, 1) and select individual 1 if $r < Q(1)$. Otherwise select individual k such that $\mathrm{Q(k-1) < r < Q(k)}$ holds, thus constituting a child in the population.

$$P(x_i) = \frac{f(x_i)}{\sum_{J=1}^{I} f(x_j)} \tag{15}$$

$$Q(i) = \sum_{j=1}^{i} P(x_j) \tag{16}$$

(5) Crossover: to avoid duplication of the additional code individuals in the first layer, the partial mapping crossover (PMX) is used for the additional code in this layer, and the downstream variables of the offspring individuals still maintain their original correspondence, thus obtaining two new chromosomes.

(6) Variation: two positions are randomly chosen to be arranged in reverse order. The lower variable is kept constant during this process, thus ensuring genetic diversity.

A detailed description of the method and steps is shown in Algorithm 1.

Algorithm 1. Methodology and detailed steps for solving the optimal solution of the fire station location model

Step1: Initialization

Step1.1: Input the parameters of the fire station siting problem, including the distance matrix d_{ij}, the coverage capacity of each class of fire station c_j, the maximum coverage S_j, the fire risk value of the demand point a_i, the maximum number of iterations I_{max}, the crossover rate p_c, the variance rate p_m, and the population size N, etc.

Step1.2: Initialize the number of iterations, $I = 0$.

Step2: Generate the first generation of populations

Step2.1: Randomly generate the initial population;

Step2.2: Coding the initial population using dual structure coding;

Step2.3: calculate the fitness function $f(x_i)$ for each individual of the population.

Step3: Identify the next generation of populations

Step3.1: Use the roulette mechanism to inherit the individual with the largest fitness function to the next generation, and calculate the probability $P_{(x_i)}$ of the inherited individual and the cumulative probability $Q_{(i)}$ of each individual.

Step3.2: Generate the next generation of evolved populations after crossover mutation. Make $I = I + 1$. Go to Step2.3 to iteratively calculate the adaptation value.

Step4: Terminate the judgment. When $I > I_{max}$, terminate the current algorithm iteration and output the current solution set.

Analysis of the Case

Information about the Case

The city selected for the example of this paper is located in the North China Plain, on the north bank of the lower reaches of the Yellow River, with a flat terrain. The land area of the city is about 4,188km. The ground elevation of the main urban area ranges from 2.5 to 7 meters, with a small difference in elevation. Except for the southeast, most of the geology is more suitable for construction and there is a lot of available land.

Currently, there are seven fire stations within the City, including one tertiary fire station, four secondary fire stations, and two primary fire stations. Basic information on the locations of the existing fire stations is shown in Table 1, and the fire station layout is shown in Figure 5:

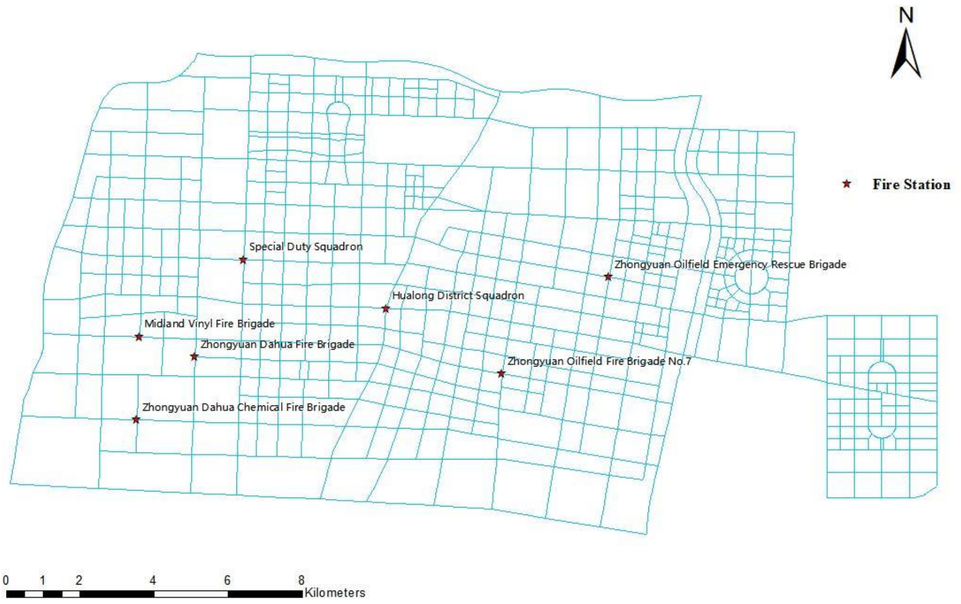

Fig 5. Schematic layout of existing fire station locations

Table 1. Basic information on existing fire stations in urban areas

No.	Name of fire station	Types of fire	floor space	Number	Number of

		station	（m^2）	of staff	vehicles
1	Hualong District Squadron	tertiary fire station	10000	35	7
2	Special Duty Squadron	secondary fire station	5000	21	4
3	Zhongyuan Oilfield Fire Brigade No.7	secondary fire station	3400	16	3
4	Midland Vinyl Fire Brigade	secondary fire station	3400	16	3
5	Zhongyuan Dahua Chemical Fire Brigade	secondary fire station	3400	15	3
6	Zhongyuan Dahua Fire Brigade	primary fire stations	2000	10	1
7	Zhongyuan Oilfield Emergency Rescue Brigade	primary fire stations	2000	11	1

As can be seen from the figure, the current layout of fire stations in the main city is relatively centralized, with two fire stations located in the center of the main city, and five primary fire stations and secondary fire stations scattered in the southwest and northeast of the neighborhood. The number of fire stations in the city is statistically small and does not cover all geographical areas. Some areas cannot be reached within the required time after the occurrence of a fire, so there is a need to optimize the location of fire stations.

According to the rescue force statistics of the city's fire stations and the recent planning documents, the selected area is divided into a sub-area by using the road intersections as the candidate facility points. Then the center of each sub-area is used as the demand point, and 13 new fire station locations are planned. The locations of the demand points and facility points are shown in Figure 8 after processing with ArcGIS.

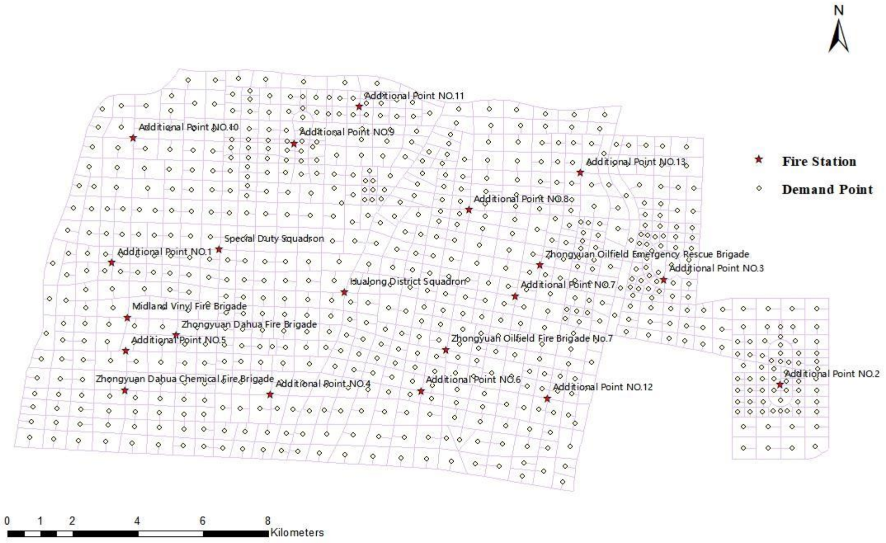

Fig 6. Schematic diagram of the planned location layout of facility points and

demand points

Results of the case

The 735 areas in the city were divided by fire risk level, and 142 high-risk areas, 438 medium-risk areas, and 155 low-risk areas were obtained. The center of gravity of the grid is used as the center of mass to represent each demand area, and the construction cost of the fire station for the three tiers is 20, 15, and 10 respectively. The distribution of the demand point locations is shown in Figure 7, and the locations and levels of the fire station facilities are shown in Table 2:

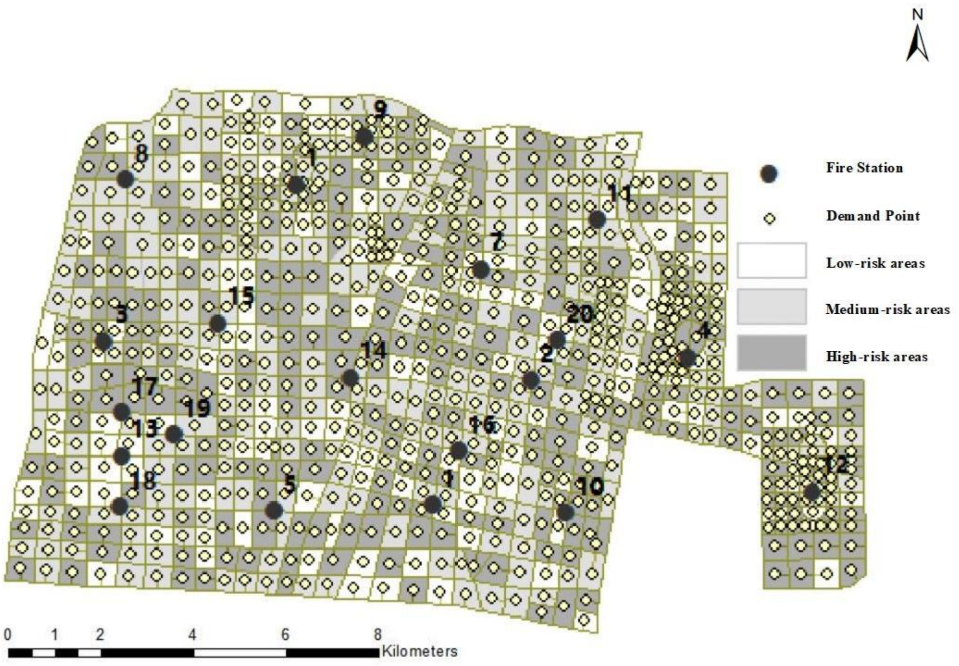

Fig 7. Distribution of demand points by level

Table 2. Location of Fire Station Facility Sites

No.	Fire Staion	Level	Fire Risk Capacity	Maximum Coverage Distance/km
1	Additional Point NO.6	3	8000	8
2	Additional Point NO.7	3	8000	8
3	Additional Point NO.1	2	5000	5
4	Additional Point NO.3	2	5000	5
5	Additional Point NO.4	2	5000	5
6	Additional Point NO.9	2	5000	5
7	Additional Point NO.8	1	3000	3
8	Additional Point NO.10	1	3000	3

9	Additional Point NO.11	1	3000	3
10	Additional Point NO.12	1	3000	3
11	Additional Point NO.13	1	3000	3
12	Additional Point NO.2	1	3000	3
13	Additional Point NO.5	1	3000	3
14	Hualong District Squadron	3	8000	8
15	Special Duty Squadron	2	5000	5
16	Zhongyuan Oilfield Fire Brigade No.7	2	5000	5
17	Midland Vinyl Fire Brigade	2	5000	5
18	Zhongyuan Dahua Chemical Fire Brigade	2	5000	5
19	Zhongyuan Dahua Fire Brigade	1	3000	3
20	Zhongyuan Oilfield Emergency Rescue Brigade	1	3000	3

The calculation platform used was Matlab R2014a. Set the population size $N = 200$, crossover probability $p_c = 0.9$, mutation probability $p_m = 0.05$, and the number of iterations to 500. Run the program and get a series of solutions by constantly changing A, B, p. When $p \leq 7$, the model is unsolved and there is no point in discussing it. Similarly, when $p > 12$, the results of the solution all fully cover the demand points, and at this point, the cost of new fire stations is getting higher and higher, and there are no realistic scenarios that can be considered. The results of solving for the values of the number of constantly changing planning sites A and retaining the original number of sites B when $p = 7\sim12$ will be listed as shown in Table 3:

Table 3. Table of solutions when $p = 7\sim12$

p	Values of A and B	target value	Cost of construction	Maximum Coverage	p	Values of A and B	target value	Cost of construction	Maximum Coverage
7	A=6，B=1	-	-	69.10%	10	A=9，B=1	59214.937	130	100%
7	A=5，B=2	-	-	68.95%	10	A=8，B=2	59317.216	120	100%

7	A=4，B=3	-	-	89.21%	10	A=7，B=3	59328.403	110	100%
7	A=3，B=4	-	-	85.53%	10	A=6，B=4	59328.403	100	100%
7	A=2，B=5	-	-	77.78%	10	A=5，B=5	59328.403	85	100%
7	A=1，B=6	-	-	70.89%	10	A=4，B=6	59328.403	70	100%
7	A=0，B=7	-	-	62.37%	10	A=3，B=7	59317.056	55	100%
8	A=7，B=1	58881.867	100	100%	11	A=10，B=1	59225.255	140	100%
8	A=6，B=2	59317.216	100	100%	11	A=9，B=2	59317.216	130	100%
8	A=5，B=3	59328.403	85	100%	11	A=8，B=3	59328.403	120	100%
8	A=4，B=4	59328.403	75	100%	11	A=7，B=4	59328.403	120	100%
8	A=3，B=5	59322.586	55	100%	11	A=6，B=5	59328.403	100	100%
8	A=2，B=6	58158.653	40	100%	11	A=5，B=6	59328.403	85	100%
8	A=1，B=7	-	-	90.19%	11	A=4，B=7	59328.403	70	100%
9	A=8，B=1	59162.472	120	100%	12	A=11，B=1	59168.243	150	100%
9	A=7，B=2	59328.403	110	100%	12	A=10，B=2	59317.216	140	100%

9	A=6，B=3	59328.403	100	100%	12	A=9，B=3	59328.403	130	100%
9	A=5，B=4	59328.403	85	100%	12	A=8，B=4	59328.403	120	100%
9	A=4，B=5	59320.557	70	100%	12	A=7，B=5	59328.403	110	100%
9	A=3，B=6	59319.748	55	100%	12	A=6，B=6	59328.403	100	100%
9	A=2，B=7	58156.019	40	100%	12	A=5，B=7	59328.403	85	100%

As can be seen from the table, when $p = 7$, the full set of possible solutions shows no solution. In this case, the maximum coverage rate is 89.21% when the siting strategy is A=4 and B=3, which means that 4 planned fire stations are selected and 3 original fire stations are retained. If covering the maximum fire risk is the main objective, when $p = 8$, there appears 7 possible solutions, of which there is no solution at this point when A=1 and B=7. The other six solutions all have 100% coverage, indicating that the solution is able to cover the entire demand point area. Options A=5, B=3, and A=4, B=4 have the same maximum fire risk covered, but the latter option is less expensive to construct than the first. When the number of fire stations required to be built by the planning authority is 8, option A=4, B=4 should be chosen. Similarly, the A=5, B=4 scenario when $p = 9$, the A=6, B=4 scenario when $p = 10$, the A=4, B=7 scenario when $p = 11$, and the A=5, B=7 scenario when p =12 are selected by the same interpretation.

If cost is regarded as the preferred consideration, based on the results of the analysis in the table above, it can be seen that when $p = 8$, the option with the lowest construction cost is A=2, B=6, but its maximum fire risk value is 1169.75 less than that of the option when A=4, B=4. Therefore, in actual construction, planners need to weigh the interactions and interactions between covering fire risk values and construction costs to select the most realistic siting option within a given budget.

Table 4 details all feasible solutions when $p = 12$. When A=5 and B=7, the value of B indicates that all of the existing fire station sites are retained, and the resulting objective function value is 59328.403. At this point, it can be seen that the last five options have the same objective function value. Comparing the cost of new facilities of these options, the last option A = 5, B = 7 costs much less to construct than the other options. The scheme was visualized using ArcGIS software and the results are shown in Figure 8:

Table 4. Trade-off solution after constantly transforming the values of A and B when p=12

Site Selection Options	1	2	3	4	5	6	7	8	9	10	11	12	13	14	15	16	17	18	19	20	objective function numerical value	New Construction Cost
A=11 B=1	1	1	1	1	1	1	1	1	0	1	1	0	1	1	0	0	0	0	0	0	59168.24	150
A=10 B=2	1	1	1	1	1	1	1	0	0	0	1	1	1	1	0	1	0	0	0	0	59317.22	140
A=9 B=3	1	1	1	1	1	1	1	0	0	0	0	1	1	1	0	1	1	0	0	0	59328.40	130
A=8 B=4	1	1	1	1	1	1	1	0	0	0	0	0	1	1	1	1	1	0	0	0	59328.40	120
A=7 B=5	1	1	1	1	1	1	0	0	0	0	0	0	1	1	1	1	1	1	0	0	59328.40	110
A=6 B=6	1	1	1	1	1	1	0	0	0	0	0	0	0	1	1	1	1	1	0	1	59328.40	100
A=5 B=7	1	1	1	1	1	0	0	0	0	0	0	0	0	1	1	1	1	1	1	1	59328.40	85

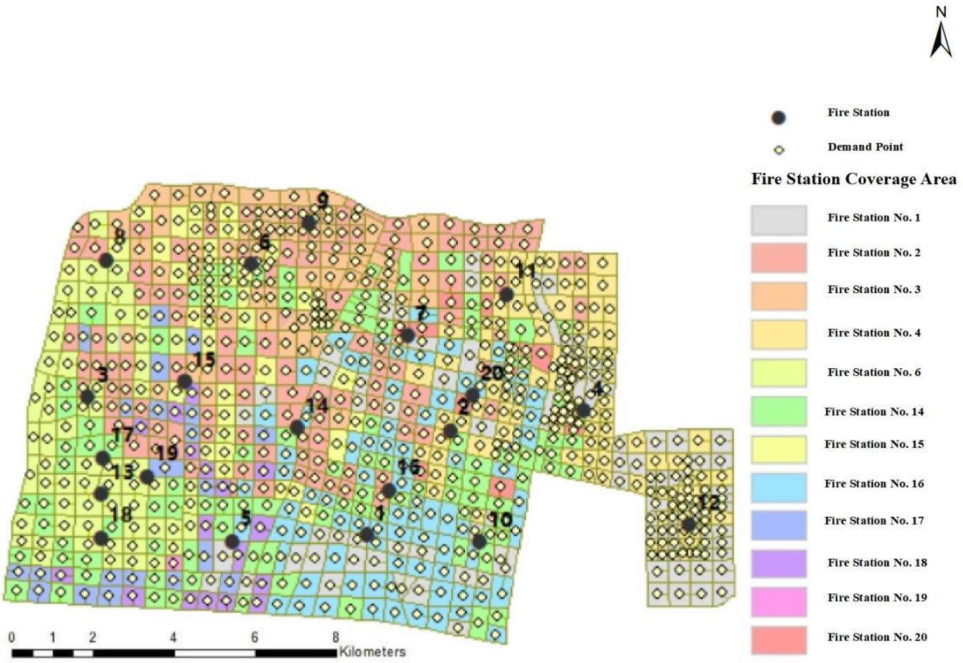

Fig 8. Fire station coverage of demand points when p=12

Next, a sensitivity analysis was performed on the element of changing fire

station takeoffs. The impact of exploring the change in maximum service coverage and retaining different numbers of fire station siting options for values of p within 1 to 20 on the results of the final model solution is shown in Figures 9 and 10:

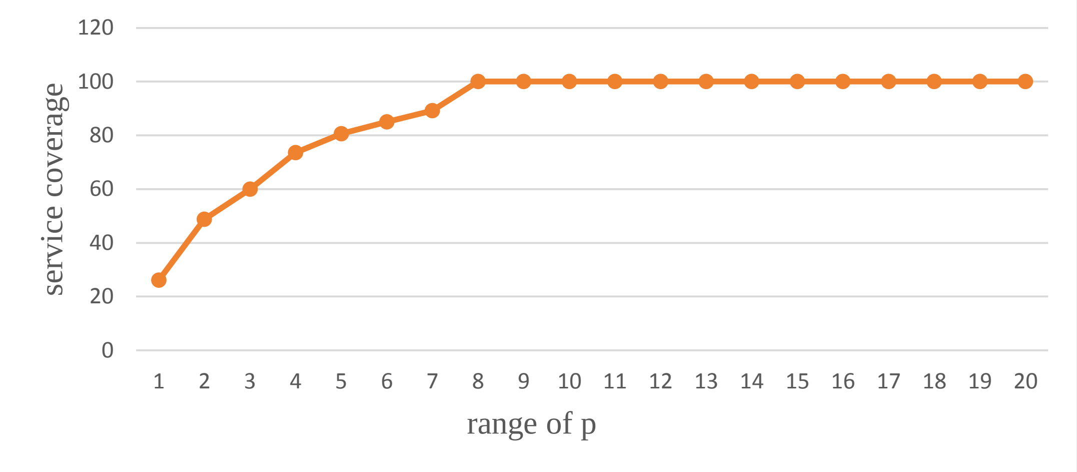

Fig 9. Variation of maximum service coverage with changing p-value

As can be seen from the above figure, although the coverage rate can reach 100% in all cases with increasing values, there is a phenomenon of partial duplication of coverage in the service area of fire stations. As the number of fire stations increases, marginal benefits diminish and construction costs increase.

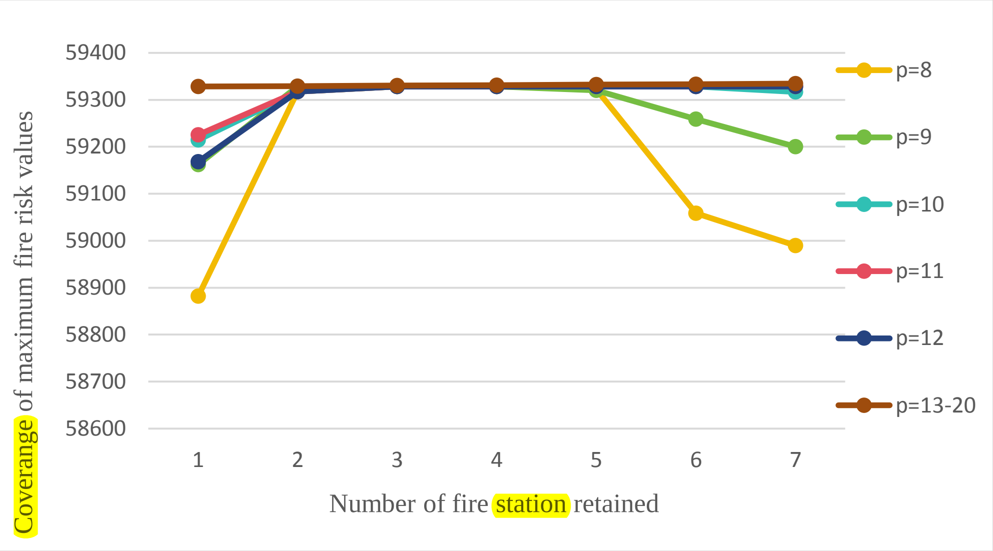

Fig 10. Variation in fire risk values and number of retained fire stations at different values

As can be seen from the above figure, when the fixed p-value is unchanged, the number of retained fire stations is increasing, then the number of new sites should be relatively reduced, and the target value of most of the siting schemes shows a trend of first increasing and then decreasing. Because of the increasing number of planned fire stations, the coverage is more and more extensive, so priority is given to the selection of fire stations with a wider coverage of the high-level fire stations, but after the target value reaches saturation, then the siting scheme gradually tends to select the fire station with a lower level and a closer distance, which leads to a reduction in the value of the coverage of the fire risk.

Conclusions

Compared with the traditional coverage siting model, this paper introduces the coverage attenuation function to reflect that different levels of fire stations provide different coverage for different demand points, and the same level of fire stations can provide more effective coverage for the demand points, which equalizes the pressure of rescue services of each level of fire stations. The model fully considers the fire risk values of different levels at the demand points, and proposes to add the existing fire station points into the planning facility point siting set to establish a hierarchical multi-objective siting model for fire stations. On the basis of using the constraint method to change the multi-objective into single-objective, the genetic algorithm is designed to solve the model, and the results are statistically and analyzed for the effects of different objectives on the decision-making scheme of fire station siting. The main conclusions are as follows:

1. Service coverage will increase with the number of new construction.

2. Although the more original fire stations that are retained, the lower the cost of siting. However, most strategies tend to fail to cover higher fire risk values and there is some leakage for fire safety issues.

3. The sensitivity analysis explores the effect of the changing number of fire stations constructed on the maximum fire risk value that can be covered by a fire station. An appropriate increase in the number of fire stations can increase the fire risk value by a certain amount, but when the number of new fire stations reaches a certain number, the risk value is saturated not only not to increase, but also to decrease.

References

1. Tian F, Lei J, Zheng X, et al. Integrating space syntax and location-allocation model for fire station location planning in a China mega city[J]. Fire, 2023, 6(2): 64.
2. Chen M, Wang K, Yuan Y, et al. A POIs based method for location optimization of urban fire station: a case study in Zhengzhou City[J]. Fire, 2023, 6(2): 58.
3. LIU N, HUANG B, CHANDRAMOULI M. Optimal siting of fire stations using GIS and ANT algorithm[J]. Journal of Computing in Civil Engineering, 20(5):361-369.
4. LI Jiapei. Comprehensive optimization model of fire station location and service[D]. Dalian University of Technology, 2017.
5. CHEN Yuehong, WU Guohao, CHEN Yu, XIA Zelong. Spatial Location Optimization of Fire Stations with Traffic Status and Urban Functional Areas[J]. Applied Spatial Analysis and Policy, 2023,16(02):771-788.
6. GUAN Wenling, ZHANG Mengjun, DONG Chengjie, XIE Jibiao. Study on multi-objective programming model for locating fire stations in chemical industry park[J]. China Safety Science Journal,2018,28(03):62-67. http://

doi.org/ 10.16265/j.cnki.issn1003-3033.2018.03.011
7. Jiang J, Zhang X, Wei R, et al. Study on location of fire stations in chemical industry parks from a public safety perspective: considering the domino effect and the identification of major hazard installations for hazardous chemicals[J]. Fire, 2023, 6(6): 218.
8. JIANG Junhao, ZHANG Xiaochun, WEI Ruichao, et al. Study on Location of Fire Stations in Chemical Industry Parks from a Public Safety Perspective: Considering the Domino Effect and the Identification of Major Hazard Installations for Hazardous Chemicals[J]. Fire,2023,6(6).
9. GH Tzeng, YW Chen. The optimal location of airport fire stations: A fuzzy multi-objective programming and revised genetic algorithm approach[J]. Transportation Planning and Technology, 1999,23(01):37-55.
10. HE Yuanhua, LI Xiang, XIONG Shenghua, CHEN Zhensong, WU Yi. Multi-objective programming model of civil airport fire station site selection based on RRCD and RADPD[J]. Computer Integrated Manufacturing Systems, 2022,28(05):1537-1548. http:// doi.org/ 10.13196/j.cims.2022.05.023.
11. DENG Yi, LI Aiqin, DOU Wei. A comparative Analysis of the Models in Urban Fire Station Layout Planning[J]. Journal of Geo-information Science, 2008(02):242-246.
12. HU Mingwei, TANG Hao. Optimization and Simulation Methods for Service Area Districting of Fire Stations[J]. China Safety Science Journal, 2008(02):242-246. http://doi.org/10.16265/j.cnki.issn1003-3033.2009.05.028.
13. CHEN Zhizong, YOU Jianxin. A Multi-objective Decision Model of Emergency Rescue Facility Location for Large-scale Emergency Incidents[J]. Journal of Management Science, 2006(04):10-14.
14. HENG Hongjun, YAN Xiaodong, WANG Fang, LI Haifeng. Research on scheduling of airport baggage transport vehicles based on multi-objective optimization[J].Computer Applications and Software, 2017,34(02):87-92+117.
15. Masood A. Badri. A multi-objective model for locating fire stations[J]. European Journal of Operational Research, 1998,110(2): 243-260.
16. ZHANG Yuanxue. Research on Fire Programming for Disastrous Hazard Source Zone Based on Multi-Objective Fuzzy Theory[D]. Tianjin University, 2007.
17. SHANG Chude. Research on Layout Optimization of Fire Rescue Station Based on Urban Fire Risk Assessment——Taking Baoding City Center as an Example[D]. Hebei Normal University, 2021.
18. YU Zhijin,XU Lan,CHEN Shuangshuang,et al. Research on urban fire station layout planning based on model intersection method[J].China Safety Science Journal, 2022,32(10):178-185. http://doi.org/10.16265/j.cnki.issn1003-3033.2022.10.2749.
19. HUO Feizhou, DONG Geli, LI Moxiao, et al. Study on location selection of linkage fire stations based on demand level and distance loss[J].China Safety Science Journal, 2022,32(03):183-193. http:// doi.org/10.16265/j.cnki.issn1003-3033.2022.03.025.

20. Moore G C, ReVelle C. The hierarchical service location problem[J]. Management science, 1982, 28(7): 775-780. http:// doi.org/ 10.1287/mnsc.28.7.775.
21. Serra D, Marianov V, ReVelle C. The maximum-capture hierarchical location problem[J]. European Journal of Operational Research, 1992, 62(3): 363-371.
22. Wang M, Jiang H, Li Y, et al. Location determination of hierarchical service facilities using a multi-layered greedy heuristic approach[J]. Engineering Optimization, 2023, 55(8): 1422-1436.
23. XIAO Junhua, HOU Yunxian. Bi-objective emergency facility location model and algorithm considering multi-level gradual coverage[J]. Soft Science, 2012, 26(12): 127-131.
