# Supplementary material for: Research on multi-objective hierarchical site selection coverage of fire station
Source: PLoS One. 2024 Dec 23;19(12):e0309731. doi: 10.1371/journal.pone.0309731 (PMC11666039; doi:10.1371/journal.pone.0309731)
Supplement: Attachment — Submitted filename: Response to Reviewers.docx [file pone.0309731.s003.docx]

Dear Editors	July 19, 2024

Enclosed is the revised version of the paper entitled “Research on multi-objective hierarchical site selection coverage of fire station”, submission ID: PONE-D-24-13730. We appreciate the very useful comments from you. We agree with your suggestions and have revised the manuscript accordingly. Below is our response to their comments resulting in several clarifications.

We tried our best to improve the manuscript and made some changes marked in red in the revised paper. We appreciate for Editors/Reviewers’ warm work earnestly and hope the corrections would be approved. Thank you very much for your comments and suggestions.

Additionally, we apologize for not making the financial disclosure part clear. This research was funded by the National Key Research and Development Program of China (No. 2020YFC1512505). The funders provided financial support and data resources on the research elements of this paper. In addition, the funders also played a role in supervision. The acknowledgements have been added in the revised manuscript.

Thanks a lot for helping us change the statement.

Best regards,

GUAN Xin

[202230210302@stu.shmtu.edu.cn](mailto:202230210302@stu.shmtu.edu.cn)

Response to Reviewer #1 Comments

**Comment 1:**

Can you explain the rationale behind using a genetic algorithm for solving the multi-objective hierarchical fire station siting problem? What specific aspects of the problem make genetic algorithms particularly suitable?

**Response:**

Thank you for your suggestion. We think it is an excellent suggestion. Based on your suggestion, we have added the fundamentals about genetic algorithms for solving the multi-objective hierarchical fire station siting problem.

The main relevant revisions are as follows:

“The site selection problem is one of the NP-hard problems, and as the size of the demand and candidate points divided in the selection area becomes larger and larger, it will take a lot of time to solve the model. Therefore, it is necessary to seek for a simpler and more effective algorithm that conforms to the actual model and is of good practical significance. Since deterministic algorithms, such as branch-and-bound and enumeration, take a long time to compute and occupy a large storage space, they are not suitable for computing large-scale optimization problems. Therefore, this paper proposes to use heuristic algorithms such as Genetic Algorithm, Particle Swarm Algorithm, Ant Colony Algorithm, Simulated Annealing Algorithm, and Greedy Algorithm, which have some differences in the optimization process and speed of solving respectively.

Genetic algorithm generally follows the evolutionary rule of survival of the fittest to get better solutions generation by generation, so as to find the optimal solution or approximate optimal solution for the problem. The algorithm requires iterative evolution through chromosome selection, crossover, mutation, and computation of fitness values. The general process is divided into the encoding of the relevant parameters, the selection of the initial chromosome, the selection of the objective function as the fitness function to calculate the fitness value corresponding to each chromosome, updating the chromosome through crossover mutation and then cycling until the fitness value meets the requirements. Genetic algorithm and other heuristic algorithms have similarities in optimization ideas, but it is not only the ability of global search is stronger, and simple and efficient to ensure a certain accuracy. Selection, crossover, and mutation are conducted with random probability, where the probabilistic feature increases the population diversity and expands the search space. Considering the complexity of the multi-objective hierarchical fire station siting model in this paper, genetic algorithm is chosen to ensure the better solution quality of the model. The specific algorithm design is as follows:”

**Comment 2:**

In the introduction, you need to connect the state of the art to your paper goals. Please follow the literature review by a clear and concise state of the art analysis. This should clearly show the knowledge gaps identified and link them to your paper goals. Please reason both the novelty and the relevance of your paper goals. Clearly discuss what the previous studies that you are referring to. What are the Research Gaps/Contributions? Please note that the paper may not be considered further without a clear research gap and novelty of the study.

**Response:**

We sincerely thank the reviewer for your careful reading. Thanks for your suggestion. We think this is an excellent suggestion. Based on your suggestion, we have rewritten the introduction of the paper.

The main relevant revisions are as follows:

(1) The gaps in literature related to this paper:

“In 2021, Shang ^[20]^ proposed to establish an urban fire risk assessment index system from four aspects: urban area information, urban fire risk sources, historical fire disaster, and fire rescue strength, and set different fire station response times based on the assessment results and optimized the layout of urban fire stations with the help of GIS software. However, the study did not consider fire station classification and remained one-sided in its assessment of urban fire risk. In 2022, Yu et al. ^[21]^ proposed the model intersection method based on maximizing the coverage model and minimizing the number of facility points model to calculate the coverage of fire risk points of interest (POI) and the area coverage of the newly planned sites. However, the examples only consider high-risk venues and are not sufficiently universal. Huo et al. ^[22]^ used ArcGIS to simulate the distribution of fire potential and graded to establish a linkage fire station location optimization model based on demand level and distance loss, which was not graded for the coverage of fire stations.”

“Xiao et al. ^[27]^ constructed a maximum coverage site selection model by considering layer characteristics and coverage decay function. However, the study only considered the tier characteristics of facilities and did not consider the tier characteristics of demand points, which is one-sided. Yet there are few studies on hierarchical siting models that incorporate the siting of emergency facilities. This paper intends to fill this gap by combining the hierarchical siting model with the fire station siting problem to construct a more realistic siting model.”

(2) Innovations in this paper

“The innovation of this paper is using a hierarchical siting model combined with the fire station siting problem in order to optimize the current urban fire station siting and achieve scientific and reasonable planning for urban fire stations. This can provide some academic help to the current theoretical research and solutions for the siting of fire stations.”

**Comment 3:**

Literature Review has the chance to be further improved: it seems that the authors have made the retrospection. However, via the review, what issues should be addressed? What is the current specific knowledge gap? What implication can be referred to? The above questions should be answered, Authors need to propose their study and compare your paper with simulation of fire stations resources considering the downtime of machines: A case study, a gis-based crisis management using fuzzy cognitive mapping: PROMETHEE approach, a new humanitarian relief logistic network for multi-objective optimization under stochastic programming.

**Response:**

Thanks for your suggestion. We think this is an excellent suggestion. Based on your suggestion, we carefully read the two papers you mentioned and summarized them in comparison with our study.

The main relevant revisions are as follows:

“Hassan Ahmadi Choukolaei et al. ^[15]^ evaluated 30 disaster shelter sites considered by crisis management using ArcGIS. The study used the PROMETEE method in ranking relief centers separately and the fuzzy mapping method to calculate the weights of the criteria for ranking relief centers. This was used to determine the best criteria for the relief center siting problem. The site selection criteria obtained from the study and the research methodology using Arcgis provide a solid research foundation for this paper.”

“Peiman Ghasemi et al. ^[23]^ presents a scenario-based stochastic multi-objective location assignment-routing model that combines both pre- and post-disaster scenarios, solves small- and medium-scale problems using the Epsilon constraint method, and solves large-scale problems using three meta-heuristic algorithms. However, again, the study did not examine the capacity of shelters in a hierarchical manner. Unlike other disasters, the capacity of fire stations and the fire risk in the demand area are hierarchically differentiated, and the difference in the hierarchy is closely related to the siting scheme, so this paper additionally considers a hierarchical siting model based on the studies in the above literature.”

**Comment 4:**

How does the dual structure coding you used in the genetic algorithm ensure that invalid individuals are avoided? Can you provide an example to illustrate how this coding works?

**Response:**

Thanks for the comments. The explanation of dual structure coding has been added.

The main relevant revisions are as follows:

“(1)Coding: For the optimization model above, dual structure coding is applied to deal with the constraints of the hierarchical fire station siting model. Dual structure coding means that the composition of the chromosome is divided into two parts, the variable code and the additional code, as shown in Figure 4 (taking six candidate points as an example). The first layer is the additional code, which represents the serial number of the candidate points for the fire station. The second layer is the corresponding 0-1 variable, which indicates whether the fire station is selected or not. The first layer is randomly generated by using shuffling and then the second layer of variables 0-1 codes are randomly generated. The assignment of demand points must satisfy the constraints of the model so that the generation of invalid individuals can be avoided.”

**Figure 4. Chromosome coding**

**Comment 5:**

In your sensitivity analysis, how did you determine the influence of parameters on the maximum fire risk value covered by fire stations? Which parameters were found to be most critical?

**Response:**

Thanks for the comments. In fact, we have conducted parameter calibration experiments before. But we didn't decide to put it in manuscript at the beginning because of the length and focus of the article. According to your suggestion, we have added two parts of sensitivity analysis, respectively genetic algorithm parameter sensitivity analysis and model parameter sensitivity analysis in the manuscript.

The main relevant revisions are as follows:

(1)Sensitivity analysis of genetic algorithm parameter

For the performance testing experiments, we have undertaken a sensitive analysis of performance for the proposed algorithm by varying different parameters. We have chosen a full factorial design in the combinations of the factors of the proposed genetic algorithm. The considered levels of all factors are shown as: population size $N\in\{180,200,220,240,260,280\}$, crossover rate $p_{c}\in\{0.6,0.7,0.8,0.9\}$, mutation rate $p_{m}\in\{0.05,0.1,0.15,0.2\}$. All the cited factors result in a total of $6\times4\times4=96$ parameter configurations for the proposed genetic algorithm. For each configuration, genetic algorithm performs 15 times calculation on each instance. Set the maximum number of iterations as 200 and the CPU time limit for each experiment as 180s. The response variable of the experiment is the relative percentage deviation (RPD). The results of the experiment are shown in Figure 9.

**Figure 9. Means plots of genetic algorithm calibration parameters**

The resulting experiment was analyzed by means of a multi-factor analysis of variance (ANOVA) technique with the least significant difference intervals at the 95% confidence level ^[29]^. Intuitively, larger population sizes should provide a higher diversity of solution distributions. Due to the limitations of the heuristic framework and probabilistic statistics, a high population size setting is not capable of accomplishing all the iterations. So, a suitable population size needs to be chosen to ensure that the algorithm achieves optimal performance. The experimental results for crossover rate $p_{c}$ and mutation rate $p_{m}$ are more in line with general expectations. Through analyzing the results in Figure 9, we set the parameters of genetic algorithm as: $N=200$, $p_{c}=0.9$, $p_{m}=0.05$. The relevant parameter descriptions are shown in Table 4.

**Table 4. Definition of parameters**

| Parameters | Meaning |
| --- | --- |
| $d_{ij}$ | The distance matrix |
| $c_{j}$ | The coverage capacity of each class of fire station |
| $S_{j}$ | The maximum coverage |
| $a_{i}$ | The fire risk value of the demand point |
| $I_{max}$ | The maximum number of iterations, $I_{max}$=500 |
| $p_{c}$ | The crossover rate, $p_{c}=0.9$ |
| $p_{m}$ | The variance rate, $p_{m}=0.05$ |
| $N$ | The population size, $N=200$ |

(2)Sensitivity analysis of p-values

“Because the emergency disaster event itself is characterized by uncertainty, the practical application of the model should consider the influence of the model parameters on the value of the objective function when they change within a reasonable range. The most important parameter of the model in this paper is the value of the number of fire stations. In order to study the impact of the number of fire stations on the objective function in different ranges of values, and to determine the reasonableness of the parameter values, this paper proposes to use the control variable method to conduct a sensitivity analysis of the parameters. It explores the impacts on the final results of the change of the maximum service coverage and the siting options of retaining different numbers of fire stations when $p=1\sim20$.

Firstly, the maximum coverage of the fire station siting options is found by gradually increasing the p-value in steps of 1. This is used to explore the decision options for changes in the number of fire stations planned and the number of fire stations retained. The obtained service coverage is shown in Table 11:

**Table 11. Service Coverage at different p-values**

| Values of $p$ | Service Coverage（%） | Values of $p$ | Service Coverage（%） |
| --- | --- | --- | --- |
| 1 | 26.15 | 11 | 100 |
| 2 | 48.73 | 12 | 100 |
| 3 | 60.04 | 13 | 100 |
| 4 | 73.58 | 14 | 100 |
| 5 | 80.65 | 15 | 100 |
| 6 | 84.98 | 16 | 100 |
| 7 | 89.21 | 17 | 100 |
| 8 | 100 | 18 | 100 |
| 9 | 100 | 19 | 100 |
| 10 | 100 | 20 | 100 |

To analyze the above data more clearly, the variation of maximum service coverage with p-value was plotted in Figure 11.

**Figure 11. Variation of maximum service coverage with changing p-value**

From the figure, it can be seen that when $1<p<7$, the service coverage rate is increasing with the growing p value. When $p\geq8$, the service coverage rate can reach 100% in all cases. However, there is the phenomenon of partial duplication of coverage of the service area of fire stations. So, it can be concluded that as the number of fire stations increases, the marginal benefit will gradually diminish and the construction cost increases.

Next, sensitivity analysis is performed for the coverage maximum fire risk value and the number of retained fire stations for different p-values. With a step size of 1, the p-value is gradually increased, while the coverage maximum fire risk value is obtained by solving with different cases of the number of retained fire stations. Based on this, a decision is made on how to develop the planned number of fire stations and the number of retained fire stations. Table 12 gives the results for multiple sets of solutions when $p=8\sim20$.

**Table 12. Solution results when** $\boldsymbol{p=8\sim20}$

|  | Coverage of maximum fire risk | | | | | |
| --- | --- | --- | --- | --- | --- | --- |
| Number of fire stations retained | p=8 | p=9 | p=10 | p=11 | p=12 | p=13-20 |
| 1 | 58881 | 59162 | 59214 | 59225 | 59168 | 59328 |
| 2 | 59317 | 59328 | 59317 | 59317 | 59317 | 59329 |
| 3 | 59328 | 59328 | 59328 | 59328 | 59328 | 59330 |
| 4 | 59328 | 59328 | 59328 | 59328 | 59328 | 59331 |
| 5 | 59322 | 59320 | 59328 | 59328 | 59328 | 59332 |
| 6 | 59058 | 59258 | 59328 | 59328 | 59328 | 59333 |
| 7 | 58989 | 59200 | 59317 | 59328 | 59328 | 59334 |

To analyze the above data more clearly, the changes in fire risk values and the number of retained fire stations at different p-values were plotted in Figure 12.

**Figure 12. Variation in fire risk values and number of retained fire stations at different values**

Analyzing the above figure, it can be seen that when the p-value is constant, if the number of fire stations retained is increasing, then the number of new sites will be reduced accordingly, and the target values of most site selection options show a trend of first increasing and then decreasing. This is because as the number of planned fire stations continues to increase, the coverage becomes wider and wider, and the model will prioritize the fire stations at the high-rise level with wider coverage. However, when the target value reaches saturation, the site selection program at this time gradually tends to select the lower level and closer fire stations, resulting in the reduction of the coverage fire risk value. The result of this solution is also consistent with the actual situation and verifies the validity of the model.”

**Comment 6:**

How did the specific geographical and infrastructural characteristics of the selected urban area influence the model's configuration and the resulting fire station siting solutions?

**Response:**

Thanks for the comments. We apologize for not explaining this clearly.

In response to this question you have asked, this paper has gone through the process of research by communicating with relevant professionals and networking to collect data. The data, information and valid information about the selected urban areas collected in this paper are shown in the table below.

**Table　Relevant information sheets for urban areas**

| No. | Name of information | Data format | Effective information that can be accessed |
| --- | --- | --- | --- |
| 1 | The City's General Plan Site Classification Map（2020-2030） | CAD | Type of site |
| 2 | Status of fire stations | Word | Existing fire station personnel and vehicles |
| 3 | Map of the current status of fire-fighting facilities（2020） | JPG | Current status of fire station layout |
| 4 | Map of the current status of the fire escape（2020） | JPG | Current status of fire escape layout |
| 5 | Fire safety layout plan（2006-2020） | JPG | Fire safety priority areas |
| 6 | Map of the current status of the land in the center of the city（2019） | JPG | Status of land use classification in the central city |
| 7 | China Fire Safety Yearbook（2020） | Book | Fire statistics for 2020 |

Based on the above information, combined with the optimization objectives and condition requirements of this city, ArcGIS software was used to perform operations such as vectorization and spatial correction on the relevant information and maps. Determine the candidate point and demand point location on the processed layer to form the fire station analysis network of this city. Finally, the fire station siting problem analyzed in this way. When the content of the above information is different, the result of fire station location will be changed accordingly.

**Comment 7:**

How did you implement the epsilon constraint method to handle the multiple objectives in your model? What were the key challenges you encountered in this process?

**Response:**

Thanks for the comments. We explained the epsilon constraint method in detail.

The main relevant revisions are as follows:

“When solving the multi-objective site selection model, focusing on one objective often requires compromising the other objective functions at the same time since the objectives conflict with each other. This makes it difficult to appear the case that all the objectives are optimal solutions, and what is obtained is often a set of equilibrium solutions, i.e., the Pareto optimal solution set. The Pareto optimal solution set is composed of many Pareto optimal solutions ^[28]^. Therefore, in this paper, the epsilon constraint method is used to deal with the multi-objective model. The Epsilon constraint method can analyze the characteristics of each objective, select the most important one as the main objective, and the rest of the objectives are transformed into the corresponding constraints and set up a change of parameter values, so as to obtain a series of solutions by solving the single-objective model. The method is stricter on the parameter values, and the epsilon constraint method is expressed as follows:

$\min f_{1}(x),x\in D$ (12)
$s.t.\left\{\begin{matrix} f_{1}x(x)\leq\varepsilon_{2} \\ ... \\ f_{m}x(x)\leq\varepsilon_{m} \end{matrix}\right.$ （13）”

Response to Reviewer #2 Comments

**Comment 1:**

More in-depth analysis of the author's contribution of this paper in the introduction section, i would like to see more discussion of the literature so that I can clearly identify the article relates to competing ideas.

**Response:**

We sincerely thank the reviewer for your careful reading. Thanks for your suggestions. We have rewritten the introduction of the paper.

The main relevant revisions are as follows:

“Hassan Ahmadi Choukolaei et al. ^[15]^ evaluated 30 disaster shelter sites considered by crisis management using ArcGIS. The study used the PROMETEE method in ranking relief centers separately and the fuzzy mapping method to calculate the weights of the criteria for ranking relief centers. This was used to determine the best criteria for the relief center siting problem. The site selection criteria obtained from the study and the research methodology using Arcgis provide a solid research foundation for this paper.”

“In 2021, Shang ^[20]^ proposed to establish an urban fire risk assessment index system from four aspects: urban area information, urban fire risk sources, historical fire disaster, and fire rescue strength, and set different fire station response times based on the assessment results and optimized the layout of urban fire stations with the help of GIS software. However, the study did not consider fire station classification and remained one-sided in its assessment of urban fire risk. In 2022, Yu et al. ^[21]^ proposed the model intersection method based on maximizing the coverage model and minimizing the number of facility points model to calculate the coverage of fire risk points of interest (POI) and the area coverage of the newly planned sites. However, the examples only consider high-risk venues and are not sufficiently universal. Huo et al. ^[22]^ used ArcGIS to simulate the distribution of fire potential and graded to establish a linkage fire station location optimization model based on demand level and distance loss, which was not graded for the coverage of fire stations. Peiman Ghasemi et al. ^[23]^ presents a scenario-based stochastic multi-objective location assignment-routing model that combines both pre- and post-disaster scenarios, solves small- and medium-scale problems using the Epsilon constraint method, and solves large-scale problems using three meta-heuristic algorithms. However, again, the study did not examine the capacity of shelters in a hierarchical manner. Unlike other disasters, the capacity of fire stations and the fire risk in the demand area are hierarchically differentiated, and the difference in the hierarchy is closely related to the siting scheme, so this paper additionally considers a hierarchical siting model based on the studies in the above literature.”

“Xiao et al.^[27]^ constructed a maximum coverage site selection model by considering layer characteristics and coverage decay function. However, the study only considered the tier characteristics of facilities and did not consider the tier characteristics of demand points, which is one-sided. Yet there are few studies on hierarchical siting models that incorporate the siting of emergency facilities. This paper intends to fill this gap by combining the hierarchical siting model with the fire station siting problem to construct a more realistic siting model.”

**Comment 2:**

More discussion and evidence should be provided for Fig. 10.

**Response:**

Thanks for the comments. In fact, we would have carried out a detailed sensitivity analysis of the parameters. But we didn't decide to put it in manuscript at the beginning because of the length and focus of the article. More discussion and evidence of the sensitivity analysis has been added in the manuscript.

The main relevant revisions are as follows:

“Next, sensitivity analysis is performed for the coverage maximum fire risk value and the number of retained fire stations for different p-values. With a step size of 1, the p-value is gradually increased, while the coverage maximum fire risk value is obtained by solving with different cases of the number of retained fire stations. Based on this, a decision is made on how to develop the planned number of fire stations and the number of retained fire stations. Table 12 gives the results for multiple sets of solutions when $p=8\sim20$.

**Table 12. Solution results when** $\boldsymbol{p=8\sim20}$

|  | Coverage of maximum fire risk | | | | | |
| --- | --- | --- | --- | --- | --- | --- |
| Number of fire stations retained | p=8 | p=9 | p=10 | p=11 | p=12 | p=13-20 |
| 1 | 58881 | 59162 | 59214 | 59225 | 59168 | 59328 |
| 2 | 59317 | 59328 | 59317 | 59317 | 59317 | 59329 |
| 3 | 59328 | 59328 | 59328 | 59328 | 59328 | 59330 |
| 4 | 59328 | 59328 | 59328 | 59328 | 59328 | 59331 |
| 5 | 59322 | 59320 | 59328 | 59328 | 59328 | 59332 |
| 6 | 59058 | 59258 | 59328 | 59328 | 59328 | 59333 |
| 7 | 58989 | 59200 | 59317 | 59328 | 59328 | 59334 |

To analyze the above data more clearly, the changes in fire risk values and the number of retained fire stations at different p-values were plotted in Figure 12.

**Figure 12. Variation in fire risk values and number of retained fire stations at different values**

Analyzing the above figure, it can be seen that when the p-value is constant, if the number of fire stations retained is increasing, then the number of new sites will be reduced accordingly, and the target values of most site selection options show a trend of first increasing and then decreasing. This is because as the number of planned fire stations continues to increase, the coverage becomes wider and wider, and the model will prioritize the fire stations at the high-rise level with wider coverage. However, when the target value reaches saturation, the site selection program at this time gradually tends to select the lower level and closer fire stations, resulting in the reduction of the coverage fire risk value. The result of this solution is also consistent with the actual situation and verifies the validity of the model.”

**Comment 3:**

The conclusions don't tie to the discussion well and should be reconsidered. There needs to be clearer discussion of the points in the body, or the conclusions should be adjusted to better match the existing discussion.

**Response:**

Thanks for the comments. More discussions were added in the conclusions.

The main relevant revisions are as follows:

“The main conclusions are as follows:

1. By analyzing the results of the experiment, with the increase in p-value, the maximum coverage increases from a minimum of 62.37% to 100%. Therefore, it can be concluded that the service coverage increases with the increasing number of new fire stations.
2. The A=2, B=6 (i.e., build 2 new fire stations and retain the original 6 fire stations) scenario obtained from the experimental results has the lowest construction cost, but its maximum fire risk value is at a lower level. This indicates that if more original fire stations are retained, the cost of siting will be lower. However, most of the strategies tend to fail to cover higher fire risk values and there is certain leakage for fire safety issues. Therefore, in actual construction, planners need to balance the interactions between covering fire risk values and construction costs. A decision is made on the optimal siting configuration within a given budget to cover the maximum fire risk value.
3. This paper analyzes the sensitivity of the element of the value taken by the fire station and explores the effect of the number of construction of fire stations on the maximum fire risk value that can be covered by the fire station. The experimental results demonstrate that although the coverage rate can reach 100% with increasing values, there is a phenomenon of partial duplicate coverage of the fire station service area. As the number of fire stations increases, the marginal benefit decreases and the construction cost increases. So, an appropriate increase in the number of new fire stations can improve the fire risk value by a certain amount. But when that number reaches a certain amount, the risk value tends to be saturated, and not only will it not be increased, but it will also be decreased. This conclusion can provide some support for decision-making when planners design a compromise and coordinated siting plan for fire stations in a hierarchical manner.

There are also some limitations to this study. For example, the relocation of fire station locations can be considered, or additions or deletions can be made to the original hierarchy to achieve the goal of increasing the hierarchy of fire stations. This study is carried out on the basis that the original fire station locations and the tiers remain unchanged. In addition, the hierarchical fire station proposed in this paper refers to a nested model, which means that a demand point can only be covered by fire stations of the same level or higher level. It can be considered that a demand point can be covered by multiple fire stations to further equalize the rescue pressure of each fire station.”

**Comment 4:**

The references below are suggested to add to further improve the readability of this work.10.1016/i.jgsce.2024.205225:10.1063/5.0206160

**Response:**

We sincerely appreciate the valuable comments. We have checked the literature carefully and added more reference on “Inhibition performances of lithium-ion battery pack fires by fine water mist in an energy-storage cabin: A simulation study” and “Plugging methods for underground gas extraction boreholes in coal seams: A review of processes, challenges and strategies” into the INTRODUCTION part in the revised manuscript.

The main relevant revisions are as follows:

“Studies on fire station siting and layout mainly cover the areas of cities ^[1-5]^, chemical industry parks ^[6-10]^, airports ^[11-12]^, etc.”

References:

9.Lou, Zhen, et al. Plugging methods for underground gas extraction boreholes in coal seams: A review of processes, challenges and strategies.[J].Gas Science and Engineering. 2024: 205225. https://doi.org/10.1016/j.jgsce.2024.205225

10.Lou, Zhen, et al. Inhibition performances of lithium-ion battery pack fires by fine water mist in an energy-storage cabin: A simulation study[J].Physics of Fluids. 36.4 (2024). https://doi.org/10.1063/5.0206160

Response to Reviewer #3 Comments

**Comment 1:**

I suggest the authors compare the proposed model's performance with those published in the literature to reveal its robustness.

**Response:**

Thanks for your suggestion. We have conducted algorithm validation experiments before. According to your suggestion, we have added it to the manuscript.

The main relevant revisions are as follows:

“**Contrast Experiments**

To test the performance of the proposed algorithm in this study, Particle Swarm Algorithm (PSO)^[30]^, Basic Immunity Algorithm (IA)^[31]^ are chosen as comparison methods. When $p=12$, the three algorithms solved different distribution center siting results.The siting results of PSO algorithm are [1, 2, 3, 5, 6, 8, 9, 10, 14, 15, 19, 20], IA algorithm's siting results are [1, 2, 3, 4, 6, 7, 8, 11, 12, 13, 15, 16], and the siting results solved by Genetic Algorithm (GA) algorithm which is chosen in this paper are [1, 2, 3, 4, 5, 14, 15, 16, 17, 18, 19, 20]. The site selection results solved by each of the three algorithms are shown in Table 7.

**Table 7. Comparison of site selection results of three algorithms**

| Algorithm | Optimal site selection when $p=12$ | Objective function value | Cost of new construction | Number of iterations |
| --- | --- | --- | --- | --- |
| PSO | 1、2、3、5、6、8、9、10、14、15、19、20 | 58752.68 | 90 | 37 |
| IA | 1、2、3、4、6、7、8、11、12、13、15、16 | 58979.53 | 93 | 54 |
| GA | 1、2、3、4、5、14、15、16、17、18、19、20 | 59328.40 | 85 | 23 |

From the results in Table 7, the GA algorithm achieves the highest objective function value and the lowest new construction cost at the same time with the least number of iterations, which indicates that the algorithm has the strongest optimization seeking ability. Compared with PSO and IA, the objective function value is improved by 0.9% and 0.5% respectively, which indicates that this algorithm is more reasonable in solving the siting problem.

To further verify the convergence performance and robustness of the algorithms, the average and standard deviation of the objective function values of the results of each algorithm are compared. The comparison of algorithm performance metrics is shown in Table 8.

**Table 8. Comparison of performance metrics of three algorithms**

| Algorithm | Average | Standard deviation |
| --- | --- | --- |
| PSO | 58731 | 412 |
| IA | 58913 | 379 |
| GA | 59780 | 310 |

As can be seen from Table 8, in terms of the standard deviation index, the GA algorithm decreases by 33% and 22% compared with the other two site selection algorithms, respectively. It shows that GA algorithm can search the optimal value more stably. At the same time, the GA algorithm also has the largest average data, which indicates that the algorithm in this paper also has good performance in convergence. ”

**Comment 2:**

The article's language needs to be slightly revised. The attached file contains many suggestions and comments.

**Response:**

Thanks for your suggestions. We feel sorry for the poor writing. We have made proofreading to improve the readability of this manuscript and made some changes in the manuscript according to the attached file. The changes are marked in red in the revised paper. We sincerely appreciate your enthusiastic work and hope that the revisions will be approved.

Response to Reviewer #4 Comments

**Comment 1:**

The abstract, while informative, could be more explicit about the key findings and contributions of the research. Including specific results from the case study could enhance its impact.

**Response:**

Thanks for your suggestion.

The Abstract after revision is as follows:

Abstract

“The fire station location has essential theoretical and practical values, not only in terms of maintaining the safety of life and property, but also enriching the optimization theory of site selection problems. To study the multi-objective siting problem of fire stations, we firstly divided demand areas and fire stations into three levels to form a comprehensive hierarchical emergency coverage network covering fire risk areas. Secondly, the nodes of the original location of the fire station were added to the set of nodes of the planned construction of the fire station. By introducing the coverage attenuation function, the multi-objective level fire station location model covering the maximum fire risk value and the minimum construction cost of the fire station was established. Then, the epsilon constraint method was used to address the multi-objective model, followed by designing the genetic algorithm based on the problem characteristics for solutions. To validate the effectiveness of the proposed model and algorithm, numerical experiments were performed, which took an urban area in China as an example. The solutions indicated that although retaining more of the original fire stations reduced the siting costs, most strategies tended to fail to cover higher values of fire risk, and the service coverage increases with the number of new fire stations. Additionally, sensitivity analysis was conducted to explore the effect of different parameters on the maximum fire risk value that can be covered by a fire station. A compromise coordinated siting scheme with a hierarchy of fire stations can be obtained by solving the proposed model. It can provide decision support for related departments to develop optimal siting configurations.”

**Comment 2:**

While the model is described well, the methods, particularly the epsilon constraint method and the genetic algorithm should be detailed more comprehensively. Including pseudo-code or algorithmic steps might help readers better understand the implementation.

**Response:**

Thanks for the comments. We have added an explanation of the epsilon constraint method to the text.

The main relevant revisions are as follows:

“When solving the multi-objective site selection model, focusing on one objective often requires compromising the other objective functions at the same time since the objectives conflict with each other. This makes it difficult to appear the case that all the objectives are optimal solutions, and what is obtained is often a set of equilibrium solutions, i.e., the Pareto optimal solution set. The Pareto optimal solution set is composed of many Pareto optimal solutions ^[28]^. Therefore, in this paper, the epsilon constraint method is used to deal with the multi-objective model. The Epsilon constraint method can analyze the characteristics of each objective, select the most important one as the main objective, and the rest of the objectives are transformed into the corresponding constraints and set up a change of parameter values, so as to obtain a series of solutions by solving the single-objective model. The method is stricter on the parameter values, and the epsilon constraint method is expressed as follows:

$\min f_{1}(x),x\in D$ (12)
$s.t.\left\{\begin{matrix} f_{1}x(x)\leq\varepsilon_{2} \\ ... \\ f_{m}x(x)\leq\varepsilon_{m} \end{matrix}\right.$ （13）”

In addition, we have added the flowchart of genetic algorithms to the text. Please refer to comment 5 for details.

**Comment 3:**

The sensitivity analysis is mentioned but not elaborated upon. A more detailed analysis of how different parameters affect the outcomes of the model would be beneficial.

**Response:**

Thanks for the comments. We added more discussion and evidence of the sensitivity analysis in the manuscript.

The main relevant revisions are as follows:

“**Sensitivity analysis**

Because the emergency disaster event itself is characterized by uncertainty, the practical application of the model should consider the influence of the model parameters on the value of the objective function when they change within a reasonable range. The most important parameter of the model in this paper is the value of the number of fire stations. In order to study the impact of the number of fire stations on the objective function in different ranges of values, and to determine the reasonableness of the parameter values, this paper proposes to use the control variable method to conduct a sensitivity analysis of the parameters. It explores the impacts on the final results of the change of the maximum service coverage and the siting options of retaining different numbers of fire stations when $p=1\sim20$.

Firstly, the maximum coverage of the fire station siting options is found by gradually increasing the p-value in steps of 1. This is used to explore the decision options for changes in the number of fire stations planned and the number of fire stations retained. The obtained service coverage is shown in Table 11:

**Table 11. Service Coverage at different p-values**

| Values of $p$ | Service Coverage（%） | Values of $p$ | Service Coverage（%） |
| --- | --- | --- | --- |
| 1 | 26.15 | 11 | 100 |
| 2 | 48.73 | 12 | 100 |
| 3 | 60.04 | 13 | 100 |
| 4 | 73.58 | 14 | 100 |
| 5 | 80.65 | 15 | 100 |
| 6 | 84.98 | 16 | 100 |
| 7 | 89.21 | 17 | 100 |
| 8 | 100 | 18 | 100 |
| 9 | 100 | 19 | 100 |
| 10 | 100 | 20 | 100 |

To analyze the above data more clearly, the variation of maximum service coverage with p-value was plotted in Figure 11.

**Figure 11. Variation of maximum service coverage with changing p-value**

From the figure, it can be seen that when $1<p<7$, the service coverage rate is increasing with the growing p value. When $p\geq8$, the service coverage rate can reach 100% in all cases. However, there is the phenomenon of partial duplication of coverage of the service area of fire stations. So it can be concluded that as the number of fire stations increases, the marginal benefit will gradually diminish and the construction cost increases.

Next, sensitivity analysis is performed for the coverage maximum fire risk value and the number of retained fire stations for different p-values. With a step size of 1, the p-value is gradually increased, while the coverage maximum fire risk value is obtained by solving with different cases of the number of retained fire stations. Based on this, a decision is made on how to develop the planned number of fire stations and the number of retained fire stations. Table 12 gives the results for multiple sets of solutions when $p=8\sim20$.

**Table 12. Solution results when** $\boldsymbol{p=8\sim20}$

|  | Coverage of maximum fire risk | | | | | |
| --- | --- | --- | --- | --- | --- | --- |
| Number of fire stations retained | p=8 | p=9 | p=10 | p=11 | p=12 | p=13-20 |
| 1 | 58881 | 59162 | 59214 | 59225 | 59168 | 59328 |
| 2 | 59317 | 59328 | 59317 | 59317 | 59317 | 59329 |
| 3 | 59328 | 59328 | 59328 | 59328 | 59328 | 59330 |
| 4 | 59328 | 59328 | 59328 | 59328 | 59328 | 59331 |
| 5 | 59322 | 59320 | 59328 | 59328 | 59328 | 59332 |
| 6 | 59058 | 59258 | 59328 | 59328 | 59328 | 59333 |
| 7 | 58989 | 59200 | 59317 | 59328 | 59328 | 59334 |

To analyze the above data more clearly, the changes in fire risk values and the number of retained fire stations at different p-values were plotted in Figure 12.

**Figure 12. Variation in fire risk values and number of retained fire stations at different values**

Analyzing the above figure, it can be seen that when the p-value is constant, if the number of fire stations retained is increasing, then the number of new sites will be reduced accordingly, and the target values of most site selection options show a trend of first increasing and then decreasing. This is because as the number of planned fire stations continues to increase, the coverage becomes wider and wider, and the model will prioritize the fire stations at the high-rise level with wider coverage. However, when the target value reaches saturation, the site selection program at this time gradually tends to select the lower level and closer fire stations, resulting in the reduction of the coverage fire risk value. The result of this solution is also consistent with the actual situation and verifies the validity of the model.”

**Comment 4:**

Clearly define all symbols and terms used in the mathematical model, as part of the mathematical notations, This clarity will assist readers who may not be familiar with specific methodologies.

**Response:**

Thanks for the comments. The definitions of variables were rewritten.

The main relevant revisions are as follows:

**Table 1. Definition of variables**

| Variables | Meaning |
| --- | --- |
| $I$ | The set of demand points, $\forall i\in I$ |
| $J$ | The set of fire station planning candidates, $\forall j\in J$ |
| $K$ | The level set of demand points, $K=\left\{ 1,2,3 \right\}$ |
| $H$ | The set of fire station levels, $H=\left\{ 1,2,3 \right\}$ |
| $k_{i}$ | The level corresponding to demand point $i$, $k_{i}\in K$ |
| $h_{j}$ | The level corresponding to facility point $j$, $h_{j}\in H$ |
| $a_{i}$ | The value of fire risk at demand point $i$ |
| $d_{ij}$ | The distance from $i$ to $j$ |
| $S_{j}$ | The maximum distance that the demand point is covered by the fire station$j$, $d_{ij}\leq S_{j}$ |
| $f_{ij}\left( d_{ij} \right)$ | The coverage provided by facility point $j$ for demand point $i$ |
| $q$ | The number of existing fire stations |
| $p$ | The number of fire stations that need to be built |
| $c_{j}$ | The capacity of the facility point $j$ |
| $\Omega$ | The collection of existing fire stations |

**Comment 5:**

While the use of genetic algorithms is noted, providing more detailed steps or a flowchart would be helpful. This could include how the algorithm optimizes the siting process and handles constraints. Further, the variables in the equations should be explicit and explained.

**Response:**

Thanks for the comments. We have added the flowchart of genetic algorithms to the text.

The main relevant revisions are as follows:

**Figure 5. Genetic Algorithm Flowchart**

In addition, we have rewritten the definitions of the variables in the equations.

**Table 4. Definition of parameters**

| Parameters | Meaning |
| --- | --- |
| $d_{ij}$ | The distance matrix |
| $c_{j}$ | The coverage capacity of each class of fire station |
| $S_{j}$ | The maximum coverage |
| $a_{i}$ | The fire risk value of the demand point |
| $I_{max}$ | The maximum number of iterations, $I_{max}$=500 |
| $p_{c}$ | The crossover rate, $p_{c}=0.9$ |
| $p_{m}$ | The variance rate, $p_{m}=0.05$ |
| $N$ | The population size, $N=200$ |

**Comment 6:**

Figures and tables should be better visualized and readable, Most of the figures are not very visible. Their quality and clarity need improvement, Figures 5, 6 and 8, more especially should be more legible, with clearer labels and a better distinction between existing and planned facilities.

**Response:**

Thanks for the comments. We are sorry for our carelessness. The quality of figure 5 (Now is figure 6), figure 6 (Now is figure 7) and figure 8 (Now is figure 10) have been improved.

The main relevant revisions are as follows:

**Figure 6. Schematic layout of existing fire station locations**

**Figure 7. Schematic diagram of the planned location layout of facility points and demand points**

**Figure 10. Fire station coverage of demand points when p=12**

**Comment 7:**

The explanation of the algorithm steps can be further simplified, For instance, the discussion on the fitness function and the roulette selection mechanism could be presented more clearly to ensure it is accessible to readers who may not be familiar with genetic algorithms.

**Response:**

Thanks for the comments. We have simplified the explanation of the algorithm steps and added an explanation of the fitness function and roulette selection mechanism to the text.

The main relevant revisions are as follows:

**Algorithm 1. Methodology and detailed steps for solving the optimal solution of the fire station location model**

Step1: Initialization

Step1.1: Input parameters, including $d_{ij}$, $c_{j}$, $S_{j}$, $a_{i}$, $I_{max}$, $p_{c}$, $p_{m}$ $N$, etc.

Step1.2: Initialize the number of iterations, $I=0$.

Step2: Generate the first generation of populations

Step2.1: Randomly generate the initial population.

Step2.2: Coding the initial population using dual structure coding.

Step2.3: Calculate the fitness function $f(x_{i})$ for each individual.

Step3: Identify the next generation of populations

Step3.1: Use the roulette mechanism and calculate the probability $P_{(x_{i})}$ of the inherited individual and the cumulative probability $Q_{(i)}$ of each individual.

Step3.2: Generate the next generation. Make $I=I+1$. Go to Step2.3 to iteratively calculate the adaptation value.

Step4: Terminate the judgment. When $I>I_{max}$, terminate the current algorithm iteration and output the current solution set.

“(3)Adaptation value function: After the initial population is formed, each chromosome in the population is evaluated by the fitness function of the population, which is used as a criterion to select the optimal solution. Observing the model gives that for each chromosome, $a_{i}$ is known, and the value of $f_{ij}\left( d_{ij} \right)$ can be derived from $d_{ij}$. In addition, equation (2) is for the maximum value, so the fitness of the chromosome is defined as the inverse of the objective function:

 （16）

(4) Selection: based on the previous step, individuals with large fitness function values are calculated, and these superior chromosomes are selected to produce the next generation of offspring, while the remaining individuals are operated according to the following roulette mechanism. For the selection of chromosomes, direct copy selection, randomized permutation selection, roulette selection, random sampling selection, one-to-one survivor selection, and tournament selection are generally adopted. Among them, the roulette method is chosen in this paper. The basic idea is that the probability of being selected among individuals is proportional to the size of their fitness value. By calculating the probability of each individual being inherited to the next generation, to determine whether the individual is inherited to the next generation.

The specific method of operation is: Separately, the fitness value of each individual is calculated as $f$. Where $I$ is the population size, the probability of inheriting to the next individual is $P_{(x_{i})}$, and the cumulative probability of each individual is $Q_{(i)}$. The two probability formulas are shown in equations (17) and (18). Generate a uniformly distributed random number $r$ in the interval (0, 1) and select individual 1 if $r<Q(1)$. Otherwise, select individual $k$ such that $Q(k-1)<r<Q(k)$ holds, thus constituting a child in the population.

 （17）

 （18）”

**Comment 8:**

The manuscript briefly mentions parameters like population size (N), crossover rate (pc), and mutation probability(pm). However, it lacks a discussion on how these parameters are chosen and their impact on the algorithm's performance. A sensitivity analysis or guidelines on parameter selection would be beneficial.

**Response:**

Thanks for the comments. According to your suggestions, we have added two parts of sensitivity analysis, respectively genetic algorithm parameter sensitivity analysis and model parameter sensitivity analysis in the manuscript.

The main relevant revisions are as follows:

For the performance testing experiments, we have undertaken a sensitive analysis of performance for the proposed algorithm by varying different parameters. We have chosen a full factorial design in the combinations of the factors of the proposed genetic algorithm. The considered levels of all factors are shown as: population size $N\in\{180,200,220,240,260,280\}$, crossover rate $p_{c}\in\{0.6,0.7,0.8,0.9\}$, mutation rate $p_{m}\in\{0.05,0.1,0.15,0.2\}$. All the cited factors result in a total of $6\times4\times4=96$ parameter configurations for the proposed genetic algorithm. For each configuration, genetic algorithm performs 15 times calculation on each instance. Set the maximum number of iterations as 200 and the CPU time limit for each experiment as 180s. The response variable of the experiment is the relative percentage deviation (RPD). The results of the experiment are shown in Figure 9.

**Figure 9. Means plots of genetic algorithm calibration parameters**

The resulting experiment was analyzed by means of a multi-factor analysis of variance (ANOVA) technique with the least significant difference intervals at the 95% confidence level ^[29]^. Intuitively, larger population sizes should provide a higher diversity of solution distributions. Due to the limitations of the heuristic framework and probabilistic statistics, a high population size setting is not capable of accomplishing all the iterations. So, a suitable population size needs to be chosen to ensure that the algorithm achieves optimal performance. The experimental results for crossover rate $p_{c}$ and mutation rate $p_{m}$ are more in line with general expectations. Through analyzing the results in Figure 9, we set the parameters of genetic algorithm as: $N=200$, $p_{c}=0.9$, $p_{m}=0.05$. The relevant parameter descriptions are shown in Table 4.

**Table 4. Definition of parameters**

| Parameters | Meaning |
| --- | --- |
| $d_{ij}$ | The distance matrix |
| $c_{j}$ | The coverage capacity of each class of fire station |
| $S_{j}$ | The maximum coverage |
| $a_{i}$ | The fire risk value of the demand point |
| $I_{max}$ | The maximum number of iterations, $I_{max}$=500 |
| $p_{c}$ | The crossover rate, $p_{c}=0.9$ |
| $p_{m}$ | The variance rate, $p_{m}=0.05$ |
| $N$ | The population size, $N=200$ |

**Comment 9:**

There is no mention of how the algorithm's performance is validated, empirically. Including a section on empirical results, comparing the algorithm's performance with other optimization methods or providing case studies, would strengthen the manuscript.

**Response:**

Thanks for the comments. The comparative experiments about algorithm performance have been added in the revised version.

The main relevant revisions are as follows:

“**Contrast Experiments**

To test the performance of the proposed algorithm in this study, Particle Swarm Algorithm (PSO)^[30]^, Basic Immunity Algorithm (IA)^[31]^ are chosen as comparison methods. When $p=12$, the three algorithms solved different distribution center siting results.The siting results of PSO algorithm are [1, 2, 3, 5, 6, 8, 9, 10, 14, 15, 19, 20], IA algorithm's siting results are [1, 2, 3, 4, 6, 7, 8, 11, 12, 13, 15, 16], and the siting results solved by Genetic Algorithm (GA) algorithm which is chosen in this paper are [1, 2, 3, 4, 5, 14, 15, 16, 17, 18, 19, 20]. The site selection results solved by each of the three algorithms are shown in Table 7.

**Table 7. Comparison of site selection results of three algorithms**

| Algorithm | Optimal site selection when $p=12$ | Objective function value | Cost of new construction | Number of iterations |
| --- | --- | --- | --- | --- |
| PSO | 1、2、3、5、6、8、9、10、14、15、19、20 | 58752.68 | 90 | 37 |
| IA | 1、2、3、4、6、7、8、11、12、13、15、16 | 58979.53 | 93 | 54 |
| GA | 1、2、3、4、5、14、15、16、17、18、19、20 | 59328.40 | 85 | 23 |

From the results in Table 7, the GA algorithm achieves the highest objective function value and the lowest new construction cost at the same time with the least number of iterations, which indicates that the algorithm has the strongest optimization seeking ability. Compared with PSO and IA, the objective function value is improved by 0.9% and 0.5% respectively, which indicates that this algorithm is more reasonable in solving the siting problem.

To further verify the convergence performance and robustness of the algorithms, the average and standard deviation of the objective function values of the results of each algorithm are compared. The comparison of algorithm performance metrics is shown in Table 8.

**Table 8. Comparison of performance metrics of three algorithms**

| Algorithm | Average | Standard deviation |
| --- | --- | --- |
| PSO | 58731 | 412 |
| IA | 58913 | 379 |
| GA | 59780 | 310 |

As can be seen from Table 8, in terms of the standard deviation index, the GA algorithm decreases by 33% and 22% compared with the other two site selection algorithms, respectively. It shows that GA algorithm can search the optimal value more stably. At the same time, the GA algorithm also has the largest average data, which indicates that the algorithm in this paper also has good performance in convergence. ”

**Comment 10:**

The manuscript should discuss the robustness of the genetic algorithm, How does the algorithm perform under different scenarios or with varying numbers of demand points and candidate sites? Addressing these questions would provide a comprehensive understanding of the algorithm's applicability.

**Response:**

Thanks for the comments. We have added robust experiments in the revised manuscript.

The main relevant revisions are as follows:

1) Genetic algorithm parameter sensitivity analysis

For the performance testing experiments, we have undertaken a sensitive analysis of performance for the proposed algorithm by varying different parameters. We have chosen a full factorial design in the combinations of the factors of the proposed genetic algorithm. The considered levels of all factors are shown as: population size $N\in\{180,200,220,240,260,280\}$, crossover rate $p_{c}\in\{0.6,0.7,0.8,0.9\}$, mutation rate $p_{m}\in\{0.05,0.1,0.15,0.2\}$. All the cited factors result in a total of $6\times4\times4=96$ parameter configurations for the proposed genetic algorithm. For each configuration, genetic algorithm performs 15 times calculation on each instance. Set the maximum number of iterations as 200 and the CPU time limit for each experiment as 180s. The response variable of the experiment is the relative percentage deviation (RPD). The results of the experiment are shown in Figure 9.

**Figure 9. Means plots of genetic algorithm calibration parameters**

The resulting experiment was analyzed by means of a multi-factor analysis of variance (ANOVA) technique with the least significant difference intervals at the 95% confidence level ^[29]^. Intuitively, larger population sizes should provide a higher diversity of solution distributions. Due to the limitations of the heuristic framework and probabilistic statistics, a high population size setting is not capable of accomplishing all the iterations. So, a suitable population size needs to be chosen to ensure that the algorithm achieves optimal performance. The experimental results for crossover rate $p_{c}$ and mutation rate $p_{m}$ are more in line with general expectations. Through analyzing the results in Figure 9, we set the parameters of genetic algorithm as: $N=200$, $p_{c}=0.9$, $p_{m}=0.05$. The relevant parameter descriptions are shown in Table 4

**Table 4. Definition of parameters**

| Parameters | Meaning |
| --- | --- |
| $d_{ij}$ | The distance matrix |
| $c_{j}$ | The coverage capacity of each class of fire station |
| $S_{j}$ | The maximum coverage |
| $a_{i}$ | The fire risk value of the demand point |
| $I_{max}$ | The maximum number of iterations, $I_{max}$=500 |
| $p_{c}$ | The crossover rate, $p_{c}=0.9$ |
| $p_{m}$ | The variance rate, $p_{m}=0.05$ |
| $N$ | The population size, $N=200$ |

2) Computational Stability

To verify the stability of the model in this paper, four different sizes of test cases are randomly generated. Except for the difference in the number of facilities, all other parameters of the algorithms are the same. As shown in Table 10, the solution algorithm designed in this study has strong computational stability and can find the optimal solution of the problem of different scales within 269s.

**Table 10. Solution results for different case sizes**

| Number of nodes (existing fire station, candidate for new fire station) | Optimal objective function value | Cost of new construction | Computing time(s) |
| --- | --- | --- | --- |
| 10(3,7) | 46736.34 | 57 | 83.46 |
| 20(7,13) | 59328.40 | 85 | 110.35 |
| 30(14,16) | 64746.37 | 110 | 230.12 |
| 40(25,15) | 69353.78 | 143 | 268.47 |

**Comment 11:**

Further, the manuscript could benefit from additional details on the criteria used to select the 13 new facility points. Explaining the rationale behind the chosen locations would provide deeper insights into the optimization process.

**Response:**

Thanks for the comments. We apologize for not explaining this clearly.

In response to this question you have asked, this paper has gone through the process of research by communicating with relevant professionals and networking to collect data. The data, information and valid information about the selected urban areas collected in this paper are shown in the table below.

Table　Relevant information sheets for urban areas

| No. | Name of information | Data format | Effective information that can be accessed |
| --- | --- | --- | --- |
| 1 | The City's General Plan Site Classification Map（2020-2030） | CAD | Type of site |
| 2 | Status of fire stations | Word | Existing fire station personnel and vehicles |
| 3 | Map of the current status of fire-fighting facilities（2020） | JPG | Current status of fire station layout |
| 4 | Map of the current status of the fire escape（2020） | JPG | Current status of fire escape layout |
| 5 | Fire safety layout plan（2006-2020） | JPG | Fire safety priority areas |
| 6 | Map of the current status of the land in the center of the city（2019） | JPG | Status of land use classification in the central city |
| 7 | China Fire Safety Yearbook（2020） | Book | Fire statistics for 2020 |

By analyzing the above information, the City's land use, fire station rescue force statistics, and recent government planning documents were extracted from it. 13 new facility points were planned based on these criteria.

**Comment 12:**

Although the manuscript mentions using road intersections as candidate points and sub-area centers as demand points, it lacks a thorough risk assessment, Including a more detailed analysis of fire risk across different areas would strengthen the case for the proposed locations.

**Response:**

Thanks for the comments. We apologize for not explaining this issue clearly. Regarding the determination of candidate facility points and demand points, the basis of this paper is as follows:

1) Determination of Candidate Facility Points

Candidate facility points are mainly determined by two methods in previous studies. One is to determine some sites where fire stations can be built as candidate points based on urban construction; the other is to take road intersections as candidate facility points. Since the fire risk value of each place is taken into account during the model construction process in this paper, and in order to facilitate the calculation of the distance between the fire station point and the demand point, it is not necessary to choose the first method. In addition, existing fire stations are included as candidate facility points in the analyzed network.

2) Determination of demand points

In this paper, we determine the demand point according to the "accident cluster" view proposed by scholars. In other words, the selected area is divided into sub-areas according to certain rules, and the center of each sub-area is taken as the demand point. In this paper, when dividing the demand area, the fire road is used to divide the planning area, and the size of the area within the area is counted. Finally, each attribute of the demand area point (area, road, access time, fire risk value, etc.) is assigned to the attribute table of the demand point.

**Comment 13:**

A comparative analysis of the current situation versus the optimized layout would be valuable. This could include metrics such as average response time, coverage percentage, and risk mitigation before and after optimization.

**Response:**

Thanks for the comments. A comparative analysis has been made through the model validation experiments.

The main relevant revisions are as follows:

“**Model Validation**

Next, the pre-optimization and post-optimization solution results are compared at different p-values to verify that the optimization model developed in this paper is meaningful. The comparison of the results before and after optimization is shown in Table 9.

**Table 9. Comparison analysis of the results before and after optimization**

| $p$ |  | Optimal objective function value | Cost of new construction | Maximum Coverage(%) | Computing time(s) |
| --- | --- | --- | --- | --- | --- |
| 8 | Before | 57763.672 | 60 | 90 | 101.25 |
|  | After | 59322.586 | 55 | 100 | 87.59 |
| 9 | Before | 57936.427 | 60 | 93 | 127.25 |
|  | After | 59319.748 | 55 | 100 | 95.33 |
| 10 | Before | 58342.363 | 73 | 95 | 149.35 |
|  | After | 59328.403 | 70 | 100 | 100.01 |
| 11 | Before | 58523.353 | 74 | 100 | 166.53 |
|  | After | 59328.403 | 70 | 100 | 107.56 |
| 12 | Before | 59036.635 | 89 | 100 | 180.45 |
|  | After | 59328.403 | 85 | 100 | 110.35 |

By comparing the site selection results of pre-optimization and post-optimization, the quality of optimal objective function value and the new construction cost obtained after optimization are both better. When $8\leq p\leq10$, only the optimized scheme can meet the coverage rate of 100%. When $11\leq p\leq12$, both the pre- and post-optimization schemes can meet the 100% coverage. The computing time consumed after optimization is reduced by 43% on average. It is verified that the optimization model established in this paper is effective and feasible and can better solve the fire station siting problem.”

**Comment 14:**

The manuscript should include empirical results demonstrating the model's effectiveness, Simulations or historical data analysis showing improved coverage and response times would provide concrete evidence of the model's benefits.

**Response:**

Thanks for the comments. More experiments were added to discuss the model’s effectiveness in the revised manuscript.

The main relevant revisions are as follows:

**Computational Stability**

To verify the stability of the model in this paper, four different sizes of test cases are randomly generated. Except for the difference in the number of facilities, all other parameters of the algorithms are the same. As shown in Table 10, the solution algorithm designed in this study has strong computational stability and can find the optimal solution of the problem of different scales within 269s.

**Table 10. Solution results for different case sizes**

| Number of nodes (existing fire station, candidate for new fire station) | Optimal objective function value | Cost of new construction | Computing time(s) |
| --- | --- | --- | --- |
| 10(3,7) | 46736.34 | 57 | 83.46 |
| 20(7,13) | 59328.40 | 85 | 110.35 |
| 30(14,16) | 64746.37 | 110 | 230.12 |
| 40(25,15) | 69353.78 | 143 | 268.47 |

**Comment 15:**

The conclusion does not address the limitations of the study or suggest directions for future research. Including a brief discussion on these aspects would provide a more balanced and comprehensive conclusion.

**Response:**

Thanks for the comments. We have written the conclusions.

The main relevant revisions are as follows:

“The main conclusions are as follows:

1. By analyzing the results of the experiment, with the increase in p-value, the maximum coverage increases from a minimum of 62.37% to 100%. Therefore, it can be concluded that the service coverage increases with the increasing number of new fire stations.
2. The A=2, B=6 (i.e., build 2 new fire stations and retain the original 6 fire stations) scenario obtained from the experimental results has the lowest construction cost, but its maximum fire risk value is at a lower level. This indicates that if more original fire stations are retained, the cost of siting will be lower. However, most of the strategies tend to fail to cover higher fire risk values and there is certain leakage for fire safety issues. Therefore, in actual construction, planners need to balance the interactions between covering fire risk values and construction costs. A decision is made on the optimal siting configuration within a given budget to cover the maximum fire risk value.
3. This paper analyzes the sensitivity of the element of the value taken by the fire station and explores the effect of the number of construction of fire stations on the maximum fire risk value that can be covered by the fire station. The experimental results demonstrate that although the coverage rate can reach 100% with increasing values, there is a phenomenon of partial duplicate coverage of the fire station service area. As the number of fire stations increases, the marginal benefit decreases and the construction cost increases. So, an appropriate increase in the number of new fire stations can improve the fire risk value by a certain amount. But when that number reaches a certain amount, the risk value tends to be saturated, and not only will it not be increased, but it will also be decreased. This conclusion can provide some support for decision-making when planners design a compromise and coordinated siting plan for fire stations in a hierarchical manner.

There are also some limitations to this study. For example, the relocation of fire station locations can be considered, or additions or deletions can be made to the original hierarchy to achieve the goal of increasing the hierarchy of fire stations. This study is carried out on the basis that the original fire station locations and the tiers remain unchanged. In addition, the hierarchical fire station proposed in this paper refers to a nested model, which means that a demand point can only be covered by fire stations of the same level or higher level. It can be considered that a demand point can be covered by multiple fire stations to further equalize the rescue pressure of each fire station.”

**Comment 16:**

Discuss the study implications. Provide a detailed discussion of how the findings can influence fire station planning and policy-making.

**Response:**

Thanks for the comments. The implications concluded from various experiments have been provided in conclusions.

How this paper influences fire station planning and policy making is reflected in: Taking an urban area as a specific example, this paper analyzes the impact of the changes in the number of fire stations constructed and the number of original fire stations retained on the maximum fire risk value that can be covered by fire stations. Finally, a compromise coordinated siting scheme for fire station hierarchy is given. The sensitivity analysis proves the feasibility and effectiveness of the proposed model and algorithm. The government can decide the number of retained fire stations versus the number of new fire stations based on the findings of this paper to maximize the coverage of the total fire risk-weighted value while saving costs.

The main relevant revisions are as follows:

“The main conclusions are as follows:

2.The A=2, B=6 (i.e., build 2 new fire stations and retain the original 6 fire stations) scenario obtained from the experimental results has the lowest construction cost, but its maximum fire risk value is at a lower level. This indicates that if more original fire stations are retained, the cost of siting will be lower. However, most of the strategies tend to fail to cover higher fire risk values and there is certain leakage for fire safety issues. Therefore, in actual construction, planners need to balance the interactions between covering fire risk values and construction costs. A decision is made on the optimal siting configuration within a given budget to cover the maximum fire risk value.

3.This paper analyzes the sensitivity of the element of the value taken by the fire station and explores the effect of the number of construction of fire stations on the maximum fire risk value that can be covered by the fire station. The experimental results demonstrate that although the coverage rate can reach 100% with increasing values, there is a phenomenon of partial duplicate coverage of the fire station service area. As the number of fire stations increases, the marginal benefit decreases and the construction cost increases. So an appropriate increase in the number of new fire stations can improve the fire risk value by a certain amount. But when that number reaches a certain amount, the risk value tends to be saturated, and not only will it not be increased, but it will also be decreased. This conclusion can provide some support for decision-making when planners design a compromise and coordinated siting plan for fire stations in a hierarchical manner.”
